# Interdisciplinary care models for functional movement disorders: a systematic review of clinical effectiveness and implications for integrated healthcare delivery

**DOI:** 10.3389/fmed.2026.1939816

**Published:** 2026-10-09

**Authors:** Anabel Granja-Dominguez, Juan Máximo Molina-Linde, Patricia García-Sanz, María Piedad Rosario-Lozano, Juan Antonio Blasco-Amaro, Alberto Marcos Heredia-Rizo

**Affiliations:** 1 Área de Evaluación de Tecnologías Sanitarias de Andalucía (AETSA), Fundación Pública Andaluza Progreso y Salud, Seville, Spain; 2 Instituto de Biomedicina de Sevilla—IBiS, Hospitales Universitarios Virgen del Rocío y Macarena/Consejo Superior de Investigaciones Científicas (CSIC)/Universidad de Sevilla, Seville, Spain; 3 Departamento de Fisioterapia, Facultad de Enfermería, Fisioterapia y Podología, Universidad de Sevilla, Seville, Spain; 4 CTS 1110: Understanding Movement and Self in Health from Science (UMSS) Research Group, Andalusia, Spain

**Keywords:** cognitive behavioral approach, combined modality therapy methods, functional disorder, movement disorders/therapy aged, quality of life

## Abstract

**Introduction:**

Functional movement disorders (FMD) are a group of highly disabling conditions that are often managed through fragmented care, despite increasing support for interdisciplinary coordinated clinical approaches. This systematic review assessed the clinical efficacy and safety of interdisciplinary interventions in adults diagnosed with FMD.

**Methods:**

MEDLINE (Ovid), Embase, the Cochrane Library, Web of Science, CINAHL, PsycINFO, PEDro, INAHTA, trial registries, and HTA agency websites were searched from inception to October 2025. Randomized controlled trials (RCTs) comparing coordinated multidisciplinary/interdisciplinary interventions combining therapeutic components delivered by professionals from different discipline with non-coordinated or non-multidisciplinary care were included. Outcomes of interest included quality of life, motor function, mental health, and reported adverse events. Risk of bias was assessed using the Cochrane Risk of Bias-2 tool.

**Results:**

Meta-analysis was not feasible because of substantial heterogeneity across studies. Five RCTs published between 2002 and 2024, including 186 participants (105 females) were included. One study was rated as having an overall high risk of bias. Compared with control interventions, interdisciplinary interventions may improve quality of life (physical domain) and motor function in the short to medium term, although findings were inconsistent across studies. No clear benefits were found for mental health status, overall clinical assessment, or social impact. Reported adverse events were infrequent and generally mild.

**Perspective:**

This systematic review synthesizes current evidence on interdisciplinary care in FMD. While short-term functional improvements have been noted, the uncertainty regarding the sustainability of these effects and their possible psychosocial impact underscores the necessity for rigorous longitudinal research in this population.

**Systematic Review Registration:**

OSF (https://doi.org/10.17605/OSF.IO/56GYX).

## 1 Introduction

Functional movement disorders (FMD) are a heterogeneous group of conditions, within the spectrum of functional neurological disorders (FNDs), characterized by impaired movement control in the absence of identifiable structural or neurodegenerative pathology (1, 2). Beyond motor symptoms, FMD are associated with problems in attentional control, emotional processing, and interoception (1, 3). FNDs occur predominantly in young adult women (1, 4) and are the second most common reason for referral to neurology services (2). Epidemiological estimates vary considerably, with annual incidence rates of 10–22 cases per 100,000 adults and prevalence ranging from 50 to 140 per 100,000 (5, 6). In general neurology, FMD account for 1% to 9% of patients, increasing to approximately 20% in specialized movement disorders centers (1). Their socioeconomic burden is substantial, as FMD often affect working-age individuals (7), and involve a combination of chronic symptoms, disability, long-term treatment needs, and fragmented care across services (6). Together, these factors underscore the need for scalable, evidence-based management strategies (6, 8).

Clinical diagnosis relies on positive signs identified during neurological examination, particularly inconsistency and variability across daily tasks, rather than on exclusion alone (1, 2). Advances in biomarker research have helped shift clinical and societal perspectives away from outdated assumptions of malingering or purely psychogenic causation (9). Neurophysiological testing and functional neuroimaging may provide additional diagnostic support. For instance, electroencephalography and electromyography can yield findings consistent with functional myoclonus and help characterize functional tremor (4). Neuroimaging has also demonstrated alterations in brain network connectivity and task-related metabolic demands, supporting a distinct neurofunctional substrate (10). Prognosis is often unfavorable; with symptoms persisting or worsening over time and complete remission occurring in only a minority of patients (7). Motor manifestations, including tremor, weakness, and mixed motor syndromes, are accompanied by pain, fatigue, and psychological distress, all of which contribute to reduced quality of life and increased use of healthcare services (4, 7, 11).

Management of FMD has traditionally been uncoordinated, with neurology, rehabilitation, and mental health services operating in parallel rather than within structured care pathways, resulting in duplicated procedures, delays in effective treatment, and inconsistent clinical messaging (12). Multidisciplinary care is increasingly supported by advances in understanding the neurobiological mechanisms underlying these disorders, with psychological factors conceptualized as potential perpetuating contributors (1, 13). Accordingly, patient-centered treatment programs now combine specialized physiotherapy, psychological interventions, such as cognitive-behavioral therapy (CBT), pharmacological treatment when indicated (14–19), and, more recently, digital strategies (11, 17, 18), allowing comprehensive assessment and tailored, goal-directed management to improve prognosis and quality of life (12). Recently, the Spanish Society of Neurology, together with international experts in neurology and psychiatry, developed a consensus document outlining clinical recommendations for FMD management and emphasizing the need for a coordinated, evidence-based model of care (12). However, the long-term effects of multimodal interventions and the predictors of treatment response remain uncertain.

This systematic review evaluates the evidence on interdisciplinary interventions for adults with FMD, focusing on clinically relevant outcomes, including motor function, psychological health, and quality of life, as well as intervention safety based on reported adverse events

## 2 Methods

The present systematic review was conducted in accordance with the Preferred Reporting Items for Systematic Reviews and Meta-Analyses (PRISMA) 2020 statement and the Cochrane Collaboration recommendations (20). The study protocol was prospectively registered in the Open Science Framework (OSF) in September 2025: https://doi.org/10.17605/OSF.IO/56GYX There were no deviations from the registered protocol.

### 2.1 Study selection

A comprehensive literature search was performed in the following databases: CINAHL (EBSCOhost), Embase, the International Health Technology Assessment (INAHTA), MEDLINE (Ovid), PEDro, PsycINFO (EBSCOhost), the Cochrane Library, and Web of Science. Searches were conducted from inception to October 2025. Additional sources included the International Clinical Trials Registry Platform (ICTRP), Clinical Trials.gov, HTA agency websites such as the Spanish Network of HTA Agencies (from Spanish: RedETS), the National Institute for Health and Care Excellence (NICE), Canada's Drugs Agency, and the Agency for Healthcare Research and Quality), and the Trip database. Language filters for studies published in English or Spanish were applied. Full search strategies are provided in Supplementary File 1.

### 2.2 Eligibility criteria

Eligibility criteria were prespecified using the Population, Intervention, Comparator, Outcomes, and Study Design (PICOD) framework, as follows:

- Population: Adults (aged 18 years or older) with a confirmed diagnosis of FMD.
- Intervention: A multidisciplinary or interdisciplinary management approach, defined as a structured and coordinated intervention combining at least two therapeutic components delivered by professionals from different disciplines. Interventions were allowed to vary in content and intensity, provided they represented an integrated model of care rather than a single-component intervention. Pharmacological or other adjunctive treatments were considered eligible only when incorporated into a broader multidisciplinary programme.
- Comparator: Any intervention or care model without a multidisciplinary or interdisciplinary approach, including standard care, single-component interventions, non-coordinated care, and placebo/sham-based treatments.
- Outcomes: Outcomes relevant to patients and clinical-decision making were selected following the Grading of Recommendations Assessment, Development and Evaluation (GRADE) principles (21) for outcome prioritisation, e.g., quality of life, motor function, health status, psychological status, and adverse events. Additional social and economic outcomes were also considered, including cost-effectiveness, as well as patient and health professional satisfaction.
- Study design: Systematic reviews (with or without meta-analyses), HTA reports, randomized controlled trials (RCTs), and economic evaluation studies.

Narrative reviews, observational studies, letters to the editor, conference abstracts, editorials, opinion pieces, and studies conducted in pediatric populations were excluded.

### 2.3 Selection process

Study selection was conducted by two independent reviewers (AG-D and JMM-L). All records retrieved from the electronic searches were imported into Covidence, a systematic review management platform (https://www.covidence.org/), where duplicates were identified and removed. The remaining citations were screened by title and abstract, and the full texts of potentially eligible studies (and those without an abstract) were then assessed for inclusion. Reasons for exclusion at the full-text stage are documented in Supplementary File 2. Disagreements were resolved through discussion, and when consensus could not be reached, a third reviewer (PG-S) was consulted. No artificial intelligence-based functions, such as automated prioritization, machine learning–assisted screening, or sampling tools were used at any stage of the selection process.

### 2.4 Risk of bias

Two examiners (AG-D, JMM-L) independently assessed the risk of bias of the included RCTs using the Cochrane Risk of Bias-2 (RoB-2) tool, which evaluates five domains: bias arising from the randomization process (D1), bias due to deviations from intended interventions (D2), bias due to missing outcome data (D3), bias in outcome measurement (D4), and bias in selection of the reported findings (D5). Domain-level judgements were used to derive an overall rating of ‘low’ risk of bias, 'some concerns', or ‘high’ risk of bias (22).

### 2.5 Data extraction and synthesis

Data were independently extracted by two reviewers (AG-D and JMM-L) using a prespecified data extraction sheet. For each included study, the following information was collected: author (s), year of publication, country, and study design; sample size and participant characteristics, including mean age and sex distribution by study group; FMD diagnostic criteria, disease duration, and diagnostic delay; baseline mental health symptoms; race/ethnicity, educational level, and family status; details of the intervention and comparator groups; intervention completion rate; outcome measures and assessment tools; and the qualitative findings of interest.

### 2.6 Narrative synthesis of results

We initially planned to undertake a meta-analysis with studies comparable in terms of interventions, control groups, and outcome measures. However, a quantitative synthesis was not considered feasible because of substantial heterogeneity across the included studies, particularly in measurement tools, follow-up duration, participant characteristics, and intervention content. The studies used a wide range of instruments to assess quality of life, motor function, mental health, social impact, and overall clinical assessment, thereby limiting the interpretability of pooled estimates. Accordingly, findings were synthesized and summarized across studies by outcome domain.

### 2.7 Qualitative evidence on patient perspectives

To incorporate patient perspectives and better reflect the real-world acceptability of care, we conducted an additional systematic search for qualitative evidence examining the experiences, perceptions, values, and preferences of individuals with FMD receiving multimodal care. Consistent with current guidance on integrating qualitative evidence into intervention reviews, this component aimed to contextualize the effectiveness findings by identifying perceived barriers, facilitators, and implementation issues (23). We included qualitative (interviews and focus groups) and mixed-methods studies with a clearly identifiable qualitative component. Eligible studies had to explore views on the diagnostic process, treatment experiences, interactions with healthcare professionals, and the impact of FMD on daily life. Perspectives from caregivers and healthcare professionals involved in multidisciplinary management, including neurologists, physiotherapists, clinical psychologists, occupational therapists, and psychiatrists, were considered. Full search strategies are provided in Supplementary File 3.

## 3 Results

### 3.1 Study selection

Electronic and manual searches identified a total of 1,896 records of interest. Of these, five studies were finally included (24–28), none of which had been retracted. A flow diagram of the study selection process is shown in Figure 1.

**Figure 1:**
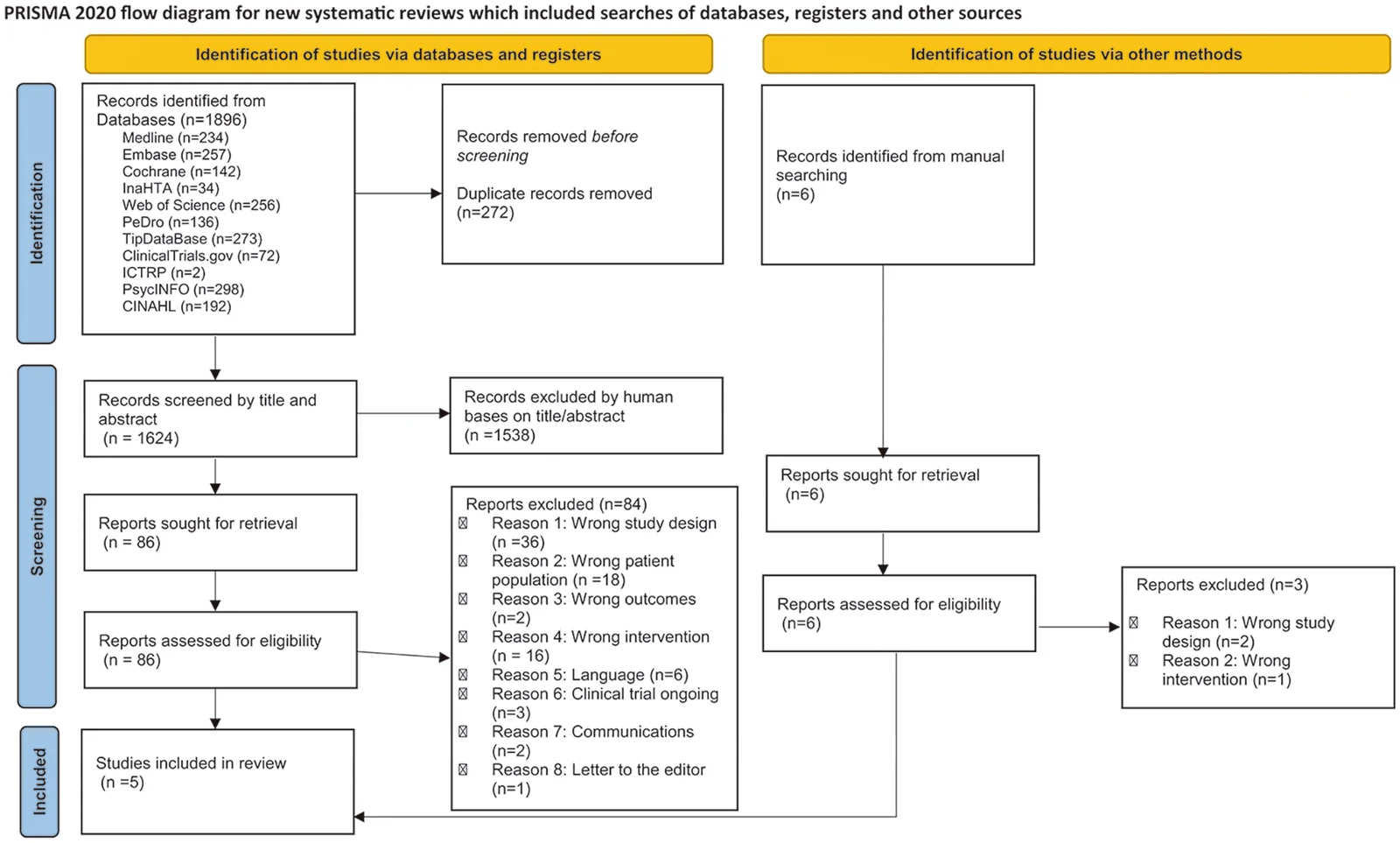
Flowchart diagram of studies identified and included in the systematic review.

### 3.2 Study characteristics

All the studies were RCTs, published between 2002 and 2024. No eligible systematic reviews, HTA reports, or economic evaluations meeting the predefined criteria were identified. The total sample comprised 186 participants (105 females, 56.5%). Most studies included patients diagnosed with FMD (24, 26–28), whereas one study involved patients with functional dystonia, a subtype of FMD (25). Diagnostic criteria varied among studies and included the phenotype-based Espay & Lang criteria (24), the Psychogenic Movement Disorders Rating Scale (PMDRS) (25), and the Diagnostic and Statistical Manual of Mental Disorders (DSM) criteria (26, 28). None of the studies provided data on race or ethnicity. The comparison groups included waiting list, standard non-coordinated care, nondirective supportive psychotherapy, CBT combined with placebo injections, and physiotherapy plus functional group therapy. Table 1 contains a comprehensive overview of the main characteristics of the included studies.

**Table 1:** Characteristics of the included studies.

Author(s), year, country, and study type	Participants, mean age: groups.	FMD diagnostic criteria (FMD duration). Diagnosis delay	Baseline mental health symptoms (mean score)	Race/ethnicity. Educational level Family status	Intervention, MDG: Duration	Comparison/s group/s: Duration	Intervention completion rate	Outcomes Assessment points
Macías-García et al, 2024 (24), Spain Single-blinded RCT	*n* = 40 (32 females), 43.5 (12.8) yrs: MDG = 20 (16 females), CG = 20 (16 females)	Phenotype-based Espay & Lang criteria (MDG, 5.6 yrs; CG, 4.6 yrs). MDG, 3.2 yrs; CG, 2.6 yrs	Anxiety (HAM-A): 20.4 Depression (BDI-II): 22.1	Race/ethnicity: N/S Educational level: basic (5), elementary (10), high school (14), bachelor or higher (11) Family status: N/S Employment status: active (9), unemployed (13), retired (2), temporal or permanent incapacity (16)	4 successive, weekly, 1-h group sessions of CBT, and 12 ambulatory 1-h individual PT sessions (based on PT consensus for FMD). Total duration: 4 wks.	Nondirective supportive psychotherapy (life stressors): 4 weekly, 1-h group sessions. Total duration: 4 wks.	Overall: 95% (38/40) MDG: 95% (19/20) CG: 95% (19/20)	Primary outcomes: Health status: EQ VAS Quality of life: EQ-5D5L, SF-36 Secondary outcomes Caregiver burden: ZCBI FMD clinical improvement: CGI-I FMD severity: sFMDRS, CGI-S T0, T1 (week 12), T2 (week 20)
Vizcarra et al, 2019 (25), US Double-blind, placebo-controlled RCT	*n* = 14^a^ (10 females), 49 (11.75) yrs: MDG = 7 (6 females), CG = 7^a^ (4 females)	PMDRS score ≥10: MDG, 21.3 (13.8); CG, 15.3 (9.6), + dystonic posturing for > 1 year. (3.3 yrs). Diagnosis delay: N/S	Anxiety (HAM-A): 19.9 Depression (HAM-D): 16.75	Race/ethnicity: N/S Educational level: N/S Family status: N/S Employment status: N/S	Onabotulinum toxinA (Botox® BoNT-A) in some muscles followed by 12 one-hour CBT sessions. Total duration: 12 wks. or until remission.	Equal amount and distribution of normal saline (as placebo) followed by 12 CBT sessions. Total duration: 12 wks. or until remission.	Overall: 71% (10/14) MDG: 86% (6/7) CG: 57% (4/7)	Primary or secondary outcomes: (N/S): FMD severity: PMDRS Mental health: HAM-A, HAM-D Quality of life: ADL, iADL T0, T1 (week 12)
Hubschmid et al, 2015 (26), Switzerland Parallel RCT	*n* = 23 (female N/S), 34,55 (3,58) yrs: MDG=11 (60% females), CG = 12 (90.91% females)	DSM-IV-TR (within 12 months) with motor or NEA symptoms assessed by experienced neurologists. Disease duration: N/S Diagnosis delay: N/S	BDI ≥ 20: 25.39 MADRS ≥15: 37.73 Mental health component of SF-36: 59.47	Race/ethnicity: N/S Educational level: N/S Family status: N/S Employment status: Work percentage before symptoms (mean):0.85 Work percentage at inclusion (mean): 0.23	2 initial and final sessions of 2.5 h (neurology consultation, psychiatric consultation and joint consultation) plus 3 to 6 intermediate sessions of psychotherapy. Total duration: 8 wks.	Standard care, involving non-coordinated consultations with neurologists and psychiatrists Total duration: 8 wks.	Overall: 91% (21/23) MDG: 91% (10/11) CG: 92% (11/12)	Primary outcomes: FMD symptoms: SDQ-20, CGI and Rankin scale Secondary outcomes: Use of medical care: number of in-patient days and outpatient visits to (a) emergency, (b) somatic clinic and (c) psychiatric follow-up. Acceptance of psychiatric referral: by asking patients Global mental health: MADRS, BDI and SF-36. Quality of life: SF-36, work ability, demand or financial aid T0, T1 (month 2), T2 (month 6), T3 (month 12)
Jordbru et al, 2014 (27), Norway Cross-over RCT	*n* = 60 (12 females), 37.55 (10.95) yrs: MDG = 31 (6 females), CG = 29 (6 females)	FMD diagnostic criteria: N/S Motor conversion disorder presenting as psychogenic gait for < 5 yrs. Disease duration: 3.3 yrs. Diagnosis delay: N/S	SF-12 (mental component): 45.1	Race/ethnicity: N/S Educational level: years of education after public school: 2 (1.8). Family status: living alone (41). Employment status: full-time (28), part-time (10), disability pension (12), regular job (5), student (5)	APA and CBT, delivered by doctors, PTs, occupational therapists, nurses and APA educators. Total duration: 3 wks.	Waiting list	Overall: 67% (40/60) (Cross over RCT, no differences between MDG and CG)	Primary outcomes: Disability: FIM and FMS Quality of life: SF-12 T0, T1 (month 1), T2 (month 12)
Moene et al, 2002 (28), The Netherlands Single-blinded RCT	*n* = 49 (34 females), 36.8 (11.31) yrs: MDG = 36 (N/S) CG = 23 (N/S)	DSM-III-R criteria (3.9 yrs). Diagnosis delay: N/S	SCL-90: 199.59	Race/ethnicity: N/S Educational level: primary (11.1%), secondary (15.6%), technical/occupational (57.8%), higher (15.5%). Family status: living alone, single or divorced (55.6%) Employment status: N/S	PT and FGT (3 times/wk. for 6 wks. and 2 times/wk. for 6 wks.) plus 8 weekly sessions of hypnosis (1 h) Total duration: 12 wks.	PT and FGT (3 times/wk. for 6 wks. and 2 times/wk. for 6 wks.) Total duration: 12 wks.	Overall: 92% (45/49) MDG: 94% (34/36) CG: 91% (21/23)	Primary outcomes: Mental health: SCL-90 Motor function: VRMC Quality of life: ICIDH, ICIDHP T0, T1 (month 1), T2 (month 2), T3 (month 8)

ADL, Katz index of independence in activities of daily living; APA, Adapted Physical Activity; CBT, Cognitive Behavioural Therapy; CG, control group; DSM-IV-TR, Diagnostic and Statistical Manual of Mental Disorders, Fourth Edition, Text Revision; EQ-5D-5L, EuroQol de 5-Dimensions 5-Levels; FGT, functional group therapy; FIM, Functional Independence Measures; FMS, Functional Mobility Scale; HAM-A, Hamilton Anxiety Rating Scale; HAM-D, Hamilton Depression Rating Scale; iADL, Lawton instrumental ADL; ICIDH, Impairments, Disabilities and Handicaps; ICIDHP, Impairments, Disabilities and Handicaps perceived problems; MADRS, Montgomery and Asberg Depression Scale; MDG, Multidisciplinary treatment group; N/S, non-specified; Physiotherapy, PT; PMDRS, Psychogenic Movement Disorders Rating Scale; PTSD, posttraumatic stress disorder; SCL-90, Symptom Checklist-90; SDQ-20, Somatoform Dissociation Questionnaire; SF-12, 12-Item Short-Form Health Survey; SF-36, Medical Outcome Study 36-Item Short Form Health Survey; VRMC, Video Rating Scale for Motor Conversion Symptoms; ZCBI, Zarit Caregiver Burden Interview. ^a^One patient in the control group withdrew from the study; their gender is not specified.

### 3.3 Risk of bias

One of the RCTs (20%) was rated as having an overall ‘high’ risk of bias (25), whereas two (40%) were rated as presenting 'some concerns' (26, 27), and the remaining two (40%) were rated with a ‘low’ risk of bias (24, 28). High risk of bias was identified in only two domains: missing outcome data (D3) and outcome measurement (D4). The randomization process (D1), deviations from intended interventions (D2), and selection of the reported result (D5) were rated as low risk of bias across all included studies (Figure 2).

**Figure 2:**
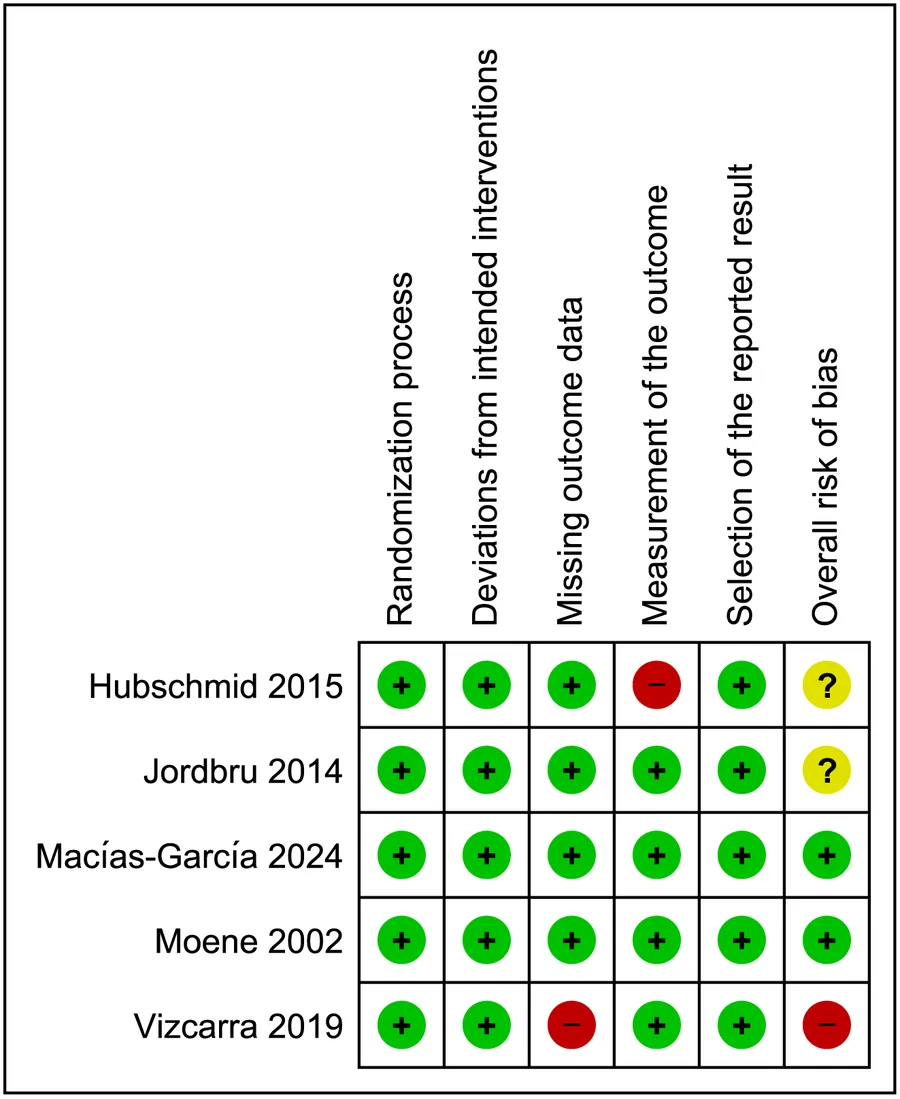
Risk of bias.

### 3.4 Safety profile

Multidisciplinary interventions showed a safety profile at least comparable to that of the comparator groups in terms of adverse events (Table 2). However, safety reporting was incomplete across studies. In two studies, adverse events were reported mainly in the control groups, including a suicide attempt (24), and a psychotic episode (25), whereas no serious adverse events were observed following the multidisciplinary approach. Unexpected hypnosis-related responses were reported in 19% of participants receiving the multidisciplinary intervention in the study by Moene et al. (28), although these were transient and not associated with major clinical consequences. Another study (26) reported no adverse events in either group, while Jordbru et al. (27) provided no information on this matter. None of the studies performed subgroup analyses by comorbidities, age, sex, or other baseline characteristics, precluding stratified estimates of the frequency of adverse events.

**Table 2:** Clinical safety of multidisciplinary interventions for individuals with functional movement disorders (reported adverse events).

Author(s), year	Type of multidisciplinary intervention	Reported adverse events	N (%) affected	Time of appearance
Macías-García et al. 2024 (24)	Psychological and physiotherapy treatment	MDG: None CG: Attempted suicide	MDG: 0 cases (0%) CG: 1 case (2.5%)	At 5 month follow-up
Vizcarra et al. 2019 (25)	Pharmacological therapy with OnabotulinumtoxinA (Botox® BoNT-A), placebo, and CBT	MDG: None CG: Psychotic episode requiring hospitalization during treatment with CBT	MDG: 0 cases (0%) CG: 1 case (7.14%)	N/R
Hubschmid et al. 2015 (26)	Psychological therapy and specialist consultations in neurology and psychiatry	MDG: None CG: None	MDG: 0 cases (0%) CG: 0 cases (0%)	N/R
Jordbru et al. 2014 (27)	Physiotherapy and psychological therapy	N/R	N/R	N/R
Moene et al. 2002 (28)	Physiotherapy and hypnotherapy	MDG: Unexpected responses to trance induction, e.g., jerking, flapping, seizures, exaggerated suggestibility, emotional outbursts and difficulty ending hypnosis). CG: None	MDG: 16 cases (19%) CG: 0 cases (0%)	During hypnosis treatment

CG, Control Group; CBT, Cognitive Behavioural Therapy; MTG, Multidisciplinary treatment group: N/R, not reported.

### 3.5 Clinical efficacy of the multidisciplinary interventions

Supplementary File 4 summarizes the reported clinical efficacy of multidisciplinary treatments, compared with comparator interventions, in individuals with FMD. Outcome measures across studies included quality of life, motor function, and mental health, as well as overall clinical assessment and social impact.

### 3.6 Quality of life

Health-related quality of life (HRQoL) was measured using several instruments, including the EuroQol-5 Dimension (EQ-5D-5L), Activities of Daily Living (ADL) and instrumental ADL (iADL), the International Classification of Impairments, Disabilities, and Handicaps (ICIDH), and the 12-Item and 36-Item Short-Form Surveys (SF-12 and SF-36). Overall, the findings suggest that multidisciplinary interventions may improve quality of life, particularly with significant differences observed in the physical domain. (SF-12 and SF-36) (24, 27). However, findings were inconsistent across studies, and long-term benefits remain unclear (25, 26, 28).

### 3.7 Motor function

Motor function was assessed using specific scales, including the Simplified Functional Movement Disorders Rating Scale (sFMDRS), Functional Mobility Scale (FMS), Functional Independence Measure (FIM), Rankin scale, and Psychogenic Movement Disorders Rating Scale (PMDRS). Overall, the included studies suggest that multidisciplinary interventions may improve motor function in the short to medium term, particularly in trials reporting improvements in the sFMDRS (24), and FMS and FIM (27). However, findings were not consistent across all studies (25, 26, 28).

### 3.8 Mental health

Mental health was evaluated using several tools, e.g., the SF-36 Mental Component Summary (SF-36 MCS), the Hamilton Anxiety (HAM-A) and Depression (HAM-D) Rating Scales, the Somatoform Dissociation Questionnaire (SDQ-20), the Beck Depression Inventory (BDI), the Montgomery and Asberg Depression Rating Scale (MADRS), the Symptom Checklist-90, and the cognitive subscale of the FIM. Findings from Hubschmid et al. (26) suggest an improvement over time in participants in the interdisciplinary treatment group for the BDI, SF-36 MCS, and SDQ-20, However, none of the included studies found significant differences between groups after treatment or at the short to medium and long-term follow-up (up to 12-months).

### 3.9 Overal clinical assessment

Overall clinical assessment was reported in two studies (24, 26), both using the Clinical Global Impression scale (CGI). Macías-García et al. (24) found significant improvements in the multidisciplinary group after treatment and at 5-month follow-up. On the contrary, Hubschmid et al. (26) observed no between-group differences at 2-, 6-, or 12-month follow-up.

### 3.10 Social impact

Social impact was assessed in a single study (24) using the Zarit Caregiver Burden Interview. No significant differences between groups were found after intervention or at the 5-month follow-up.

### 3.11 Patients perspectives with multidisciplinary interventions

Electronic and manual searches identified a total of 222 records in the search for qualitative evidence. Despite this, none of them met the eligibility criteria within the search period, mainly because they did not involve patients with FMD population or a multidisciplinary clinical management. The selection process for qualitative evidence is detailed in Figure 3.

**Figure 3:**
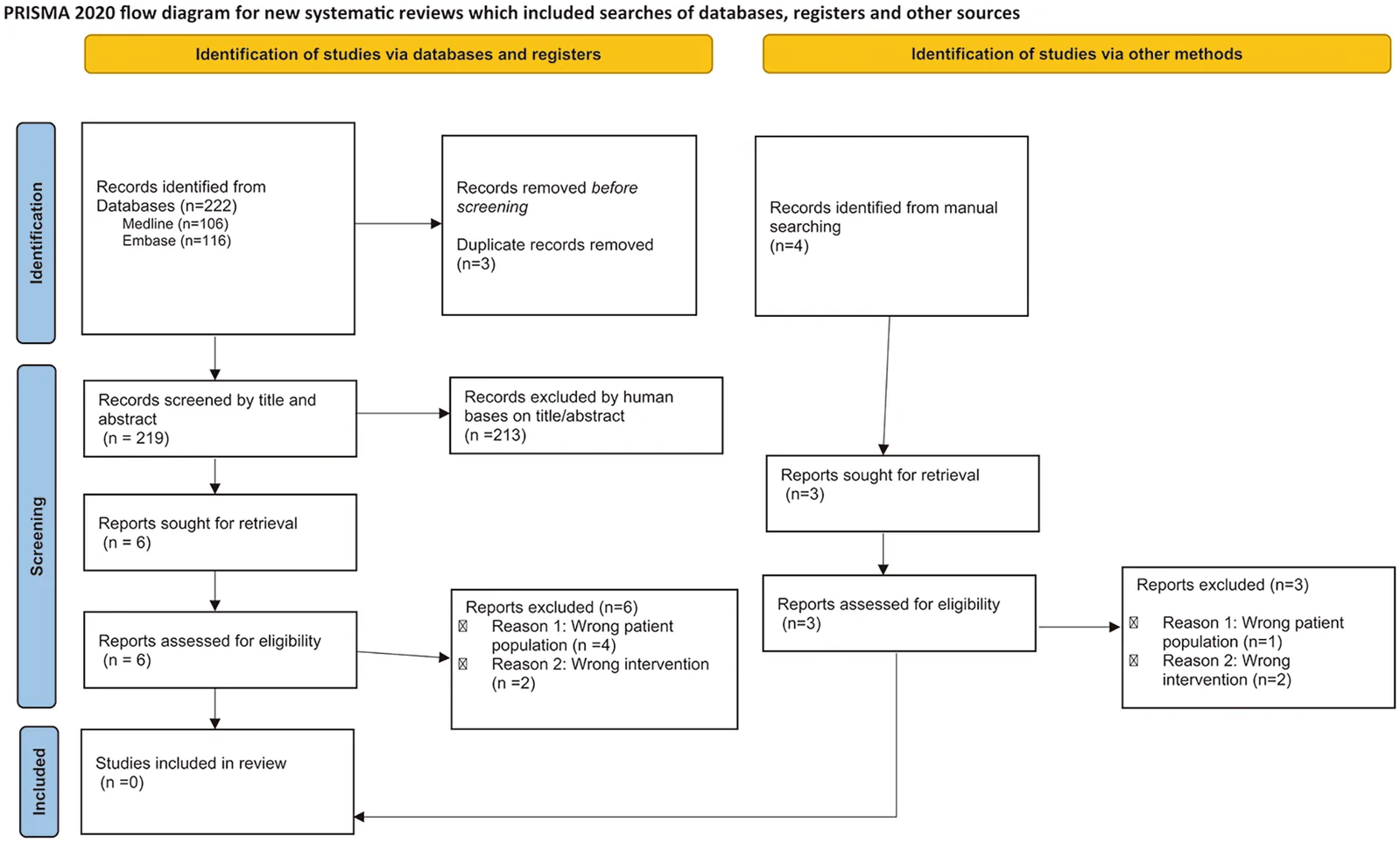
Flowchart diagram of studies identified in the qualitative review.

## 4 Discussion

The findings of this systematic review suggest that multidisciplinary or interdisciplinary interventions for individuals with FMD have a favorable safety profile and may provide modest benefits across several clinical domains, although results were heterogeneous among studies. Potential benefits were mainly observed in quality of life (physical domain) and in motor function in the short to medium term. Improvements in mental health were limited to within-group analyses. Results for overall clinical assessment were mixed, and only one study evaluated social impact. Most studies were rated as having low risk of bias or as presenting some concerns. No qualitative evidence on patient perspectives met the eligibility criteria, highlighting an important gap in understanding patient-reported experiences with multidisciplinary care in FMD. Confidence in the findings is limited by the small number of trials, modest overall sample size, heterogeneity in interventions and outcome measures, and risk-of-bias concerns in some studies.

In recent years, there has been a marked evolution in the management of FMD, with a growing body of clinical evidence supporting the use of multidisciplinary approaches (11). However, the current evidence base seems to remain limited, as only five studies met the inclusion criteria and meta-analysis was not feasible. Quality of life and motor function were assessed in all studies, although findings varied substantially, likely reflecting differences in intervention design, follow-up time points, and measurement tools. Improvements in the physical component of quality of life were reported in two studies (24, 27). Similarly, benefits in motor function were observed, particularly in the sFMDRS, as reported by Macias-García et al. (24), and in measures of mobility and autonomy (27). These positive results were not consistently replicated in the remaining studies (25, 26, 28). In this context, longer follow-up periods may be relevant, since sustained benefits may emerge over time even with lower-intensity interventions (29). Overall, even though statistically significant between-group differences were not consistently demonstrated, multidisciplinary strategies incorporating physiotherapy may still contribute to clinical improvements in quality of life and motor function-related aspects in adults with FMD.

Evidence for the impact of interdisciplinary interventions on mental health, clinical assessment and social impact remains limited. No consistent between-group differences were observed for cross-sectional measures of anxiety, depression, or general psychological functioning. However, longitudinal results from selected studies indicate that mental health benefits may appear over time, especially for depressive and somatoform symptoms (26). In line with this, broader evidence indicates that, although no single intervention has shown to be definitively effective, interdisciplinary approaches offer promising benefits (30). Additionally, integrated inpatient programmes have been associated with sustained improvements in psychological well-being over a 12-month period (16). By contrast, evidence on social impact is particularly scarce, with no studies assessing outcomes such as participation, return to work, or social autonomy. Future studies should incorporate valid instruments, e.g., the WHO Disability Assessment Schedule 2.0 (31), to provide a more comprehensive evaluation of the psychosocial effects of interdisciplinary interventions in this population.

Multidisciplinary care for FMD showed a favorable safety profile and appeared to be well tolerated by patients. Across studies, adverse events were infrequent, mild, or absent, and serious events were reported only in comparator groups (24, 25). Moene et al. (28) described transient hypnosis-related responses, with no major adverse events directly attributed to the multidisciplinary intervention. Overall, these findings support a safety profile of multidisciplinary management in FMD.

The economic implications of multidisciplinary/interdisciplinary care should also be considered. However, our review did not identify sufficient evidence to assess the cost-effectiveness of this model in FMD. One recent randomized controlled trial in patients with FMD evaluated the economic impact of a digital telemedicine intervention, providing an example of how resource use and costs can be incorporated into the evaluation of FMD interventions, although this approach is not directly comparable to the multidisciplinary/interdisciplinary care model assessed in our review (32). Similarly, evidence from broader functional neurological or functional somatic disorder populations may support developing specialised services (33). Further FMD-specific research should therefore assess the costs and resource requirements of coordinated multidisciplinary/interdisciplinary care alongside clinical outcomes.

In this review, no qualitative studies were found exploring patient perspectives on multidisciplinary or interdisciplinary care for FMD. Existing qualitative evidence from non-interventional FMD cohorts concludes that patients experience a substantial physical and emotional burden, often accompanied by feelings of abandonment and incomprehension with the healthcare system (34, 35). The current findings underscore the limited understanding of FMD and the possible lack of adequately structured care pathways, which may hinder the development of targeted and effective models of care. Furthermore, this review's lack of qualitative evidence limits understanding of patient experiences and factors affecting engagement with multidisciplinary care. Since our October 2025 search, a new qualitative study of FMD patients revealed insights into symptom changes, emotional regulation, social participation, and treatment factors like the therapeutic relationship and patient readiness. Experiences varied, with some patients facing ongoing symptoms (36). Though published after our review, this study underscores the importance of patient perspectives in evaluating such care models. Future research should include qualitative or mixed-methods approaches to better understand patient experiences, engagement, and barriers, and to improve implementation of coordinated care models.

### 4.1 Study limitation

The present findings should be interpreted considering several methodological limitations. The terminological diversity surrounding FMD reflects its evolving conceptualization and limits both cohort definition and cross-study comparability (37), thereby hampering evidence synthesis and reinforcing the need for rigorous diagnostic frameworks in search strategies and eligibility criteria. In addition, marked heterogeneity in multidisciplinary protocols, including treatment duration, session frequency, and therapeutic components, limited comparability and reproducibility across studies. The wide range of outcome measures used, together with inconsistent reporting metrics, also prevented a quantitative synthesis. The results are further constrained by the small number of RCTs, the limited description of intervention content, and the absence of subgroup analyses by age, sex, or comorbidities. Therefore, the applicability of these findings to individualized clinical practice remains limited. The certainty of evidence was not formally assessed using GRADE. Further longitudinal studies using standardized protocols, consistent outcome measures, and longer follow-ups are needed to properly inform robust clinical recommendations.

## 5 Conclusion

Coordinated multidisciplinary/interdisciplinary interventions for FMD comprising, among others, neurological, pharmacological, physiotherapeutic, and psychological components appear to be safe and may offer limited short- to medium-term benefits in quality of life (physical domain) and motor function, although findings remain exploratory and inconsistent across studies. Evidence regarding mental health, overall clinical assessment, and social or caregiver-related impact was inconsistent. These conclusions are limited by a substantial methodological heterogeneity across the small number of studies included.

## Data Availability

The original contributions presented in the study are included in the article/Supplementary Material, further inquiries can be directed to the corresponding author.
