# Supplementary material for: Interdisciplinary care models for functional movement disorders: a systematic review of clinical effectiveness and implications for integrated healthcare delivery
Source: Front Med (Lausanne). 2026 Oct 9;13:1939816. doi: 10.3389/fmed.2026.1939816 (PMC13690528; doi:10.3389/fmed.2026.1939816)
Supplement: Supplementary file 1 [file Table1.docx]

**Supplementary File 1.** Search Strategies

**Ovid MEDLINE (10/2025)**

1 exp "Conversion Disorder"/

2 ((psychogenic or functional) adj3 (motor* or movement* or neurological) adj3 (disorder* or symptom*)).ti,ab,kf.

3 (((disorder? or reaction or Hysteria or Neuros#s) adj3 Conversion) or Astasia?Abasia).tw.

4 Hysteria/

5 ((Hysterical adj1 Neuros?s) or Hysteria).tw.

6 Movement Disorders/

7 ("dyskinesia syndrome?" or "etat marbre" or "status marmoratus").tw.

8 Nervous System Diseases/px [Psychology]

9 Psychophysiologic Disorders/

10 ((psychophysiological or psychosomatic) adj3 disorder?).tw.

11 ((disorder? or hysteria? or reaction?) adj3 dissociative).tw.

12 1 or 2 or 3 or 4 or 5 or 6 or 7 or 8 or 9 or 10 or 11

13 exp Interprofessional Relations/ or Interdisciplinary Communication/ or Physician-Nurse Relations/

14 (((Interprofessional or Inter-professional or interpersonal or inter-personal or interinstitutional or inter-institutional) adj3 relation*) or (Etiquette adj1 Medical)).tw,ab,kw.

15 (multidisciplin* or multi-disciplin* or interdisciplin* or inter-disciplin* or transdisciplin* or trans-disciplin* or multiprofession* or multi-profession* or interprofession* or inter-profession* or transprofession* or trans-profession* or multispecialty or multi-specialty or multimodal* or multi-modal* or multifacet* or multi-facet* or multioccupation* or multi-occupation* or interoccupation* or inter-occupation*).ti,ab,kw.

16 ((collaborat* or coordinat* or co-ordinat* or cooperative or co-operative or continu* or shared or network or joint or integrat* or stepped or systematic or augment* or enhance*) adj3 (approach or care or team* or health* or work* or interven* or service* or model* or effort* or manag*)).ti,ab,kw.

17 exp Patient Care Team/

18 (('Health Care' or Healthcare or health or care) adj3 Team?).tw.

19 patient care management/ or exp Patient Care Planning/ or exp "delivery of health care, integrated"/

20 ((patient or nursing or goal?) adj3 care).tw.

21 ((system? or 'health care') adj3 integrated).tw.

22 Cooperative Behavior/ or Comprehensive Health Care/ 23 (((Cooperative or Compliant) adj3 Behaviors) or (Comprehensive adj3 Care)).tw.

24 13 or 14 or 15 or 16 or 17 or 18 or 19 or 20 or 21 or 22 or 23 (1192848)

25 12 and 24

26 (letter or "case report*" or "historical article*" or (comment or editorial or in vitro or news)).pt.

27 25 not 26

28 meta-analysis.pt.

29 meta-analysis/ or systematic review/ or meta-analysis as topic/ or "meta analysis (topic)"/ or "systematic review (topic)"/ or exp technology assessment, biomedical/

30 ((systematic* adj3 (review* or overview*)) or (methodologic* adj3 (review* or overview*))).ti,ab,kf,kw.

31 ((quantitative adj3 (review* or overview* or synthes*)) or (research adj3 (integrati* or overview*))).ti,ab,kf,kw.

32 ((integrative adj3 (review* or overview*)) or (collaborative adj3 (review* or overview*)) or (pool* adj3 analy*)).ti,ab,kf,kw.

33 (data synthes* or data extraction* or data abstraction*).ti,ab,kf,kw.

34 (handsearch* or hand search*).ti,ab,kf,kw.

35 (mantel haenszel or peto or der simonian or dersimonian or fixed effect* or latin square*).ti,ab,kf,kw.

36 (met analy* or metanaly* or technology assessment* or HTA or HTAs or technology overview* or technology appraisal*).ti,ab,kf,kw.

37 (meta regression* or metaregression*).ti,ab,kf,kw.

38 (meta-analy* or metaanaly* or systematic review* or biomedical technology assessment* or bio-medical technology assessment*).mp,hw.

39 (medline or cochrane or pubmed or medlars or embase or cinahl).ti,ab,hw. (403575)

40 (cochrane or (health adj2 technology assessment) or evidence report).jw.

41 (comparative adj3 (efficacy or effectiveness)).ti,ab,kf,kw.

42 (outcomes research or relative effectiveness).ti,ab,kf,kw.

43 ((indirect or indirect treatment or mixed-treatment) adj comparison*).ti,ab,kf,kw.

44 28 or 29 or 30 or 31 or 32 or 33 or 34 or 35 or 36 or 37 or 38 or 39 or 40 or 41 or 42 or 43

45 27 and 44

46 animals/ not (animals/ and humans/)

47 45 not 46

48 Randomized Controlled Trials as Topic/

49 Randomized controlled trial/

50 Random Allocation/

51 Double Blind Method/

52 Single Blind Method/

53 Clinical trial/

54 Clinical trial, phase i.pt.

55 Clinical trial, phase ii.pt.

56 Clinical trial, phase iii.pt.

57 Clinical trial, phase iv.pt.

58 Controlled clinical trial.pt.

59 Randomized controlled trial.pt.

60 Multicenter study.pt.

61 Multicenter study.pt.

62 Clinical trial.pt.

63 (clinical adj trial$).tw.

64 ((singl$ or doubl$ or treb$ or tripl$) adj (blind$3 or mask$3)).tw.

65 placebos/

66 placebo$.tw.

67 Randomly allocated.tw.

68 (allocated adj2 random$).tw.

69 48 or 49 or 50 or 51 or 52 or 53 or 54 or 55 or 56 or 57 or 58 or 59 or 60 or 61 or 62 or 63 or 64 or 65 or 66 or 67 or 68

70 27 and 69

71 animals/ not (animals/ and humans/)

72 70 not 71

73 47 or 72

Date 16/10/2025: **studies retrieved = 234**

**Embase (10/2025)**

| #1 | 'conversion disorder'/exp |
| --- | --- |
| #2 | ((psychogenic OR functional) NEAR/3 (motor* OR movement* OR neurological) NEAR/3 (disorder* OR symptom*)):ti,ab,kw |
| #3 | (((disorder$ OR reaction OR hysteria OR neuros?s) NEAR/3 conversion):ti,ab) OR astasia$abasia:ti,ab |
| #4 | 'hysteria'/de |
| #5 | ((hysterical NEAR/1 neuros?s):ti,ab) OR hysteria:ti,ab |
| #6 | 'motor dysfunction'/de |
| #7 | "dyskinesia syndrome$":ti,ab OR 'etat marbre':ti,ab OR 'status marmoratus':ti,ab |
| #8 | 'neurologic disease'/de AND 'psychology'/de |
| #9 | 'psychosomatic disorder'/de |
| #10 | ((psychophysiological OR psychosomatic) NEAR/3 disorder$):ti,ab |
| #11 | ((disorder$ OR hysteria$ OR reaction$) NEAR/3 dissociative):ti,ab |
| #12 | #1 OR #2 OR #3 OR #4 OR #5 OR #6 OR #7 OR #8 OR #9 OR #10 OR #11 |
| #13 | 'public relations'/exp OR 'interdisciplinary communication'/de OR 'doctor nurse relation'/de |
| #14 | (((interprofessional OR 'inter professional' OR interpersonal OR 'inter personal' OR interinstitutional OR 'inter institutional') NEAR/3 relation*):ti,ab,kw) OR ((etiquette NEAR/1 medical):ti,ab,kw) |
| #15 | multidisciplin*:ti,ab,kw OR 'multi disciplin*':ti,ab,kw OR interdisciplin*:ti,ab,kw OR 'inter disciplin*':ti,ab,kw OR transdisciplin*:ti,ab,kw OR 'trans disciplin*':ti,ab,kw OR multiprofession*:ti,ab,kw OR 'multi profession*':ti,ab,kw OR interprofession*:ti,ab,kw OR 'inter profession*':ti,ab,kw OR transprofession*:ti,ab,kw OR 'trans profession*':ti,ab,kw OR multispecialty:ti,ab,kw OR 'multi specialty':ti,ab,kw OR multimodal*:ti,ab,kw OR 'multi modal*':ti,ab,kw OR multifacet*:ti,ab,kw OR 'multi facet*':ti,ab,kw OR multioccupation*:ti,ab,kw OR 'multi occupation*':ti,ab,kw OR interoccupation*:ti,ab,kw OR 'inter occupation*':ti,ab,kw |
| #16 | ((collaborat* OR coordinat* OR 'co ordinat*' OR cooperative OR 'co operative' OR continu* OR shared OR network OR joint OR integrat* OR stepped OR systematic OR augment* OR enhance*) NEAR/3 (approach OR care OR team* OR health* OR work* OR interven* OR service* OR model* OR effort* OR manag*)):ti,ab,kw |
| #17 | 'patient care team'/exp |
| #18 | (('health care' OR healthcare OR health OR care) NEAR/3 team$):ti,ab |
| #19 | 'patient care'/de OR 'patient care planning'/exp OR 'integrated health care system'/exp |
| #20 | ((patient OR nursing OR goal$) NEAR/3 care):ti,ab |
| #21 | ((system$ OR 'health care') NEAR/3 integrated):ti,ab |
| #22 | 'cooperation'/de OR 'health care'/de |
| #23 | (((cooperative OR compliant) NEAR/3 behaviors):ti,ab) OR ((comprehensive NEAR/3 care):ti,ab) |
| #24 | #13 OR #14 OR #15 OR #16 OR #17 OR #18 OR #19 OR #20 OR #21 OR #22 OR #23 |
| #25 | #12 AND #24 |
| #26 | 'conference abstract'/it OR 'conference paper'/it OR 'short survey'/it OR 'abstract report'/exp |
| #27 | #25 NOT #26 |
| #28 | 'meta analysis'/exp OR 'meta analysis topic'/de OR 'meta analysis (topic)'/exp OR 'systematic review'/exp OR 'systematic review (topic)'/exp OR 'biomedical technology assessment'/exp |
| #29 | ((systematic* NEAR/3 (review* OR overview*)):ti,ab,kw) OR ((methodologic* NEAR/3 (review* OR overview*)):ti,ab,kw) |
| #30 | ((quantitative NEAR/3 (review* OR overview* OR synthes*)):ti,ab,kw) OR ((research NEAR/3 (integrati* OR overview*)):ti,ab,kw) |
| #31 | ((integrative NEAR/3 (review* OR overview*)):ti,ab,kw) OR ((collaborative NEAR/3 (review* OR overview*)):ti,ab,kw) OR ((pool* NEAR/3 analy*):ti,ab,kw) |
| #32 | (data NEAR/1 (synthes* OR extraction* OR abstraction*)):ti,ab,kw |
| #33 | handsearch*:ti,ab,kw OR ((hand NEAR/1 search*):ti,ab,kw) |
| #34 | 'matel haenszel':ti,ab,kw OR peto:ti,ab,kw OR 'der simonian':ti,ab,kw OR 'dersimonian':ti,ab,kw OR ((fixed NEAR/1 effect*):ti,ab,kw) OR ((latin NEAR/1 square*):ti,ab,kw) |
| #35 | 'meta analy*':ti,ab,kw OR metanaly*:ti,ab,kw OR 'technology assessment*':ti,ab,kw OR hta:ti,ab,kw OR htas:ti,ab,kw OR ((technology NEAR/1 (overview* OR appraisal*)):ti,ab,kw) |
| #36 | 'meta regression*':ti,ab,kw OR metaregression*:ti,ab,kw |
| #37 | medline:ti,ab,kw OR cochrane:ti,ab,kw OR pubmed:ti,ab,kw OR medlars:ti,ab,kw OR embase:ti,ab,kw OR cinahl:ti,ab,kw |
| #38 | cochrane:jt OR ((health NEAR/2 'technology asseessment'):jt) OR 'evidence report':jt |
| #39 | (comparative NEAR/3 (efficacy OR effectiveness)):ti,ab,kw |
| #40 | 'outcomes reseach':ti,ab,kw OR 'relative effectiveness':ti,ab,kw |
| #41 | ((indirect OR 'indirect treatment' OR 'mixed-treatment') NEAR/1 comparison*):ti,ab,kw |
| #42 | #28 OR #29 OR #30 OR #31 OR #32 OR #33 OR #34 OR #35 OR #36 OR #37 OR #38 OR #39 OR #40 OR #41 |
| #43 | #27 AND #42 |
| #44 | #43 AND [embase]/lim NOT ([embase]/lim AND [medline]/lim) AND [humans]/lim |
| #45 | 'clinical trial'/de OR 'randomized controlled trial'/de OR 'controlled clinical trial'/de OR 'multicenter study'/de OR 'phase 3 clinical trial'/de OR 'phase 4 clinical trial'/de OR 'randomization'/exp OR 'single blind procedure'/de OR 'double blind procedure'/de OR 'crossover procedure'/de OR 'placebo'/de OR 'prospective study'/de OR "randomi?ed controlled trial$":ti,ab OR rct:ti,ab OR ((random$ NEAR/2 allocat$):ti,ab) OR "single blind$":ti,ab OR "double blind$":ti,ab OR (((treble OR triple) NEAR/1 blind$):ti,ab) OR placebo$:ti,ab |
| #46 | #27 AND #45 |
| #47 | #46 AND [embase]/lim NOT ([embase]/lim AND [medline]/lim) AND [humans]/lim |
| #48 | #44 OR #47 |

Date 16/10/2025: **studies retrieved = 257**

**Cochrane (10/2025)**

#1	[mh "Conversion Disorder"]

#2	((psychogenic or functional) NEAR/3 (motor* or movement* or neurological) NEAR/3 (disorder* or symptom*)):ti,ab,kw

#3	(((disorder? or reaction or Hysteria or Neuros#s) NEAR/3 Conversion) or Astasia?Abasia):ti,ab

#4	[mh Hysteria]

#5	((Hysterical NEAR/1 Neuros?s) or Hysteria):ti,ab

#6	[mh ^"Movement Disorders"]

#7	("dyskinesia syndrome?" or "etat marbre" or "status marmoratus"):ti,ab

#8	[mh ^"Nervous System Diseases"/px]

#9	[mh "Psychophysiologic Disorders"]

#10	((psychophysiological or psychosomatic) NEAR/3 disorder?):ti,ab

#11	((disorder? or hysteria? or reaction?) NEAR/3 dissociative):ti,ab

#12	#1 or #2 or #3 or #4 or #5 or #6 or #7 or #8 or #9 or #10 or #11

#13	[mh "Interprofessional Relations"] or [mh "Interdisciplinary Communication"] or [mh "Physician-Nurse Relations"]

#14	(((Interprofessional or Inter-professional or interpersonal or inter-personal or interinstitutional or inter-institutional) NEAR/3 relation*) or (Etiquette NEAR/1 Medical)):ti,ab,kw

#15	(multidisciplin* or multi-disciplin* or interdisciplin* or inter-disciplin* or transdisciplin* or trans-disciplin* or multiprofession* or multi-profession* or interprofession* or inter-profession* or transprofession* or trans-profession* or multispecialty or multi-specialty or multimodal* or multi-modal* or multifacet* or multi-facet* or multioccupation* or multi-occupation* or interoccupation* or inter-occupation*):ti,ab,kw

#16	((collaborat* or coordinat* or co-ordinat* or cooperative or co-operative or continu* or shared or network or joint or integrat* or stepped or systematic or augment* or enhance*) NEAR/3 (approach or care or team* or health* or work* or interven* or service* or model* or effort* or manag*)):ti,ab,kw

#17	[mh "Patient Care Team"]

#18	(('Health Care' or Healthcare or health or care) NEAR/3 Team?):ti,ab

#19	[mh ^"patient care management"] or [mh "Patient Care Planning"] or [mh "delivery of health care, integrated"]

#20	((patient or nursing or goal?) NEAR/3 care):ti,ab

#21	((system? or 'health care') NEAR/3 integrated):ti,ab

#22	[mh "Cooperative Behavior"] or [mh "Comprehensive Health Care"]

#23	(((Cooperative or Compliant) NEAR/3 Behaviors) or (Comprehensive NEAR/3 Care)):ti,ab

#24	#13 or #14 or #15 or #16 or #17 or #18 or #19 or #20 or #21 or #22 or #23

#25	#12 AND #24

Date 16/10/2025: **studies retrieved = 142**

**InaHTA (10/2025)**

1	"Conversion Disorder"[mh]

2	((psychogenic or functional) AND (motor* or movement* or neurological) AND (disorder* or symptom*))[Title] OR ((psychogenic or functional) AND (motor* or movement* or neurological) AND (disorder* or symptom*))[abs]

3	(((disorder* or reaction or Hysteria or Neuros*) AND Conversion) or Astasia* Abasia)[title] OR (((disorder* or reaction or Hysteria or Neuros*) AND Conversion) or Astasia* Abasia)[abs]

4	"Hysteria"[mh]

5	((Hysterical AND Neuros*) or Hysteria)[title] OR ((Hysterical AND Neuros*) or Hysteria)[abs]

6	"Movement Disorders"[mh]

7	("dyskinesia syndrome*" or "etat marbre" or "status marmoratus")[title] OR ("dyskinesia syndrome*" or "etat marbre" or "status marmoratus")[abs]

8	"Nervous System Diseases"[mh]

9	"Psychophysiologic Disorders"[mh]

10	((psychophysiological or psychosomatic) AND disorder*)[title] OR ((psychophysiological or psychosomatic) AND disorder*)[abs]

11	((disorder* or hysteria* or reaction*) AND dissociative)[title] OR ((disorder* or hysteria* or reaction*) AND dissociative)[abs]

12	#11 OR #10 OR #9 OR #8 OR #7 OR #6 OR #5 OR #4 OR #3 OR #2 OR #1

13	"Interprofessional Relations"[mh] OR "Interdisciplinary Communication"[mh] OR "Physician-Nurse Relations"[mh]

14	(((Interprofessional or Inter-professional or interpersonal or inter-personal or interinstitutional or inter-institutional) AND relation*) or (Etiquette AND Medical))[title] OR (((Interprofessional or Inter-professional or interpersonal or inter-personal or interinstitutional or inter-institutional) AND relation*) or (Etiquette AND Medical))[abs]

15	(multidisciplin* or multi-disciplin* or interdisciplin* or inter-disciplin* or transdisciplin* or trans-disciplin* or multiprofession* or multi-profession* or interprofession* or inter-profession* or transprofession* or trans-profession* or multispecialty or multi-specialty or multimodal* or multi-modal* or multifacet* or multi-facet* or multioccupation* or multi-occupation* or interoccupation* or inter-occupation*)[title] OR (multidisciplin* or multi-disciplin* or interdisciplin* or inter-disciplin* or transdisciplin* or trans-disciplin* or multiprofession* or multi-profession* or interprofession* or inter-profession* or transprofession* or trans-profession* or multispecialty or multi-specialty or multimodal* or multi-modal* or multifacet* or multi-facet* or multioccupation* or multi-occupation* or interoccupation* or inter-occupation*)[abs]

16	((collaborat* or coordinat* or co-ordinat* or cooperative or co-operative or continu* or shared or network or joint or integrat* or stepped or systematic or augment* or enhance*) AND (approach or care or team* or health* or work* or interven* or service* or model* or effort* or manag*))[title] OR ((collaborat* or coordinat* or co-ordinat* or cooperative or co-operative or continu* or shared or network or joint or integrat* or stepped or systematic or augment* or enhance*) AND (approach or care or team* or health* or work* or interven* or service* or model* or effort* or manag*))[abs]

17	"Patient Care Team"[mh]

18	(('Health Care' or Healthcare or health or care) AND Team*)[title] OR (('Health Care' or Healthcare or health or care) AND Team*)[abs]

19	"Patient Care Management"[mh] OR "Patient Care Planning"[mh] OR "Delivery of Health Care, Integrated"[mh]

20	((patient or nursing or goal*) AND care)[title] OR ((patient or nursing or goal*) AND care)[abs]

21	((system* or 'health care') AND integrated)[title] OR ((system* or 'health care') AND integrated)[abs]

22	"Competitive Behavior"[mh] OR "Comprehensive Health Care"[mh]

23	(((Cooperative or Compliant) AND Behaviors) or (Comprehensive AND Care))[title] OR (((Cooperative or Compliant) AND Behaviors) or (Comprehensive AND Care))[abs]

24	#23 OR #22 OR #21 OR #20 OR #19 OR #18 OR #17 OR #16 OR #15 OR #14 OR #13

25	#24 AND #12

Date 16/10/2025: **studies retrieved = 34**

**Web of Science (SCI) (10/2025)**

1 TI="Conversion Disorder" OR AB="Conversion Disorder"

2 TI=((psychogenic or functional) NEAR/3 (motor* or movement* or neurological) NEAR/3 (disorder* or symptom*)) OR AB=((psychogenic or functional) NEAR/3 (motor* or movement* or neurological) NEAR/3 (disorder* or symptom*))

3 TI=(((disorder* or reaction or Hysteria or Neuros*) NEAR/3 Conversion) or Astasia-Abasia) OR AB=(((disorder* or reaction or Hysteria or Neuros*) NEAR/3 Conversion) or Astasia-Abasia)

4 TI=Hysteria OR AB=Hysteria

5 TI=((Hysterical NEAR/1 Neuros*) or Hysteria) OR AB=((Hysterical NEAR/1 Neuros*) or Hysteria)

6 TI="Movement Disorders" OR AB="Movement Disorders"

7 TI=("dyskinesia syndrome*" or "etat marbre" or "status marmoratus") OR AB=("dyskinesia syndrome*" or "etat marbre" or "status marmoratus")

8 TI=("Nervous System Diseases" AND Psychology) OR AB=("Nervous System Diseases" AND Psychology)

9 TI="Pschophysiologic Disorders" OR AB="Psychophysiologic Disorders"

10 TI=((psychophysiological or psychosomatic) NEAR/3 disorder*) OR AB=((psychophysiological or psychosomatic) NEAR/3 disorder*)

11 TI=((disorder* or hysteria* or reaction*) NEAR/3 dissociative) OR AB=((disorder* or hysteria* or reaction*) NEAR/3 dissociative)

12 #11 OR #10 OR #9 OR #8 OR #7 OR #6 OR #5 OR #4 OR #3 OR #2 OR #1

13 TI=("Interprofessional Relations" or "Interdisciplinary Communication" or "Physician-Nurse Relations") OR AB=("Interprofessional Relations" or "Interdisciplinary Communication" or "Physician-Nurse Relations")

14 TI=(((Interprofessional or Inter-professional or interpersonal or inter-personal or interinstitutional or inter-institutional) NEAR/3 relation*) or (Etiquette NEAR/1 Medical)) OR AB=(((Interprofessional or Inter-professional or interpersonal or inter-personal or interinstitutional or inter-institutional) NEAR/3 relation*) or (Etiquette NEAR/1 Medical))

15 TI=(multidisciplin* or multi-disciplin* or interdisciplin* or inter-disciplin* or transdisciplin* or trans-disciplin* or multiprofession* or multi-profession* or interprofession* or inter-profession* or transprofession* or trans-profession* or multispecialty or multi-specialty or multimodal* or multi-modal* or multifacet* or multi-facet* or multioccupation* or multi-occupation* or interoccupation* or inter-occupation*) OR AB=(multidisciplin* or multi-disciplin* or interdisciplin* or inter-disciplin* or transdisciplin* or trans-disciplin* or multiprofession* or multi-profession* or interprofession* or inter-profession* or transprofession* or trans-profession* or multispecialty or multi-specialty or multimodal* or multi-modal* or multifacet* or multi-facet* or multioccupation* or multi-occupation* or interoccupation* or inter-occupation*)

16 TI=((collaborat* or coordinat* or co-ordinat* or cooperative or co-operative or continu* or shared or network or joint or integrat* or stepped or systematic or augment* or enhance*) NEAR/3 (approach or care or team* or health* or work* or interven* or service* or model* or effort* or manag*)) OR AB=((collaborat* or coordinat* or co-ordinat* or cooperative or co-operative or continu* or shared or network or joint or integrat* or stepped or systematic or augment* or enhance*) NEAR/3 (approach or care or team* or health* or work* or interven* or service* or model* or effort* or manag*))

17 TI="Patient Care Team" OR AB="Patient Care Team"

18 TI=(("Health Care" or Healthcare or health or care) NEAR/3 Team*) OR AB=(("Health Care" or Healthcare or health or care) NEAR/3 Team*) OR AB=(("Health Care" or Healthcare or health or care) NEAR/3 Team*) OR AB=(("Health Care" or Healthcare or health or care) NEAR/3 Team*)

19 TI=("patient care management" or "Patient Care Planning" or "delivery of health care") OR AB=("patient care management" or "Patient Care Planning" or "delivery of health care")

20 TI=((patient or nursing or goal*) NEAR/3 care) OR AB=((patient or nursing or goal*) NEAR/3 care)

21 TI=((system* or "health care") NEAR/3 integrated) OR AB=((system* or "health care") NEAR/3 integrated)

22 TI=("Cooperative Behavior" or "Comprehensive Health Care") OR AB=("Cooperative Behavior" or "Comprehensive Health Care")

23 TI=(((Cooperative or Compliant) NEAR/3 Behaviors) or (Comprehensive NEAR/3 Care)) OR AB=(((Cooperative or Compliant) NEAR/3 Behaviors) or (Comprehensive NEAR/3 Care))

24 #23 OR #22 OR #21 OR #20 OR #19 OR #18 OR #17 OR #16 OR #15 OR #14 OR #13

25 #24 AND #12

26 TI=(random* or blind* or allocat* or assign* or trial* or placebo* or crossover* or cross-over*) OR AB=(random* or blind* or allocat* or assign* or trial* or placebo* or crossover* or cross-over*)

27 #26 AND #25

28 TI=(systematic review) OR AB=(systematic review)

29 #25 AND #28

30 #29 OR #27

Date 16/10/2025: **studies retrieved = 256**

**PsycInfo (10/2025)**

S1 DE "Conversion Disorder" OR DE "Functional Paralysis" OR DE "Functional Visual Symptoms" OR DE "Pseudocyesis" OR DE "Pseudodementia"

S2 TI ( TI ( ((psychogenic or functional) N3 (motor* or movement* or neurological) N3 (disorder* or symptom*)) ) OR ( ((psychogenic or functional) N3 (motor* or movement* or neurological) N3 (disorder* or symptom*)) ) ) OR AB ( TI ( ((psychogenic or functional) N3 (motor* or movement* or neurological) N3 (disorder* or symptom*)) ) OR ( ((psychogenic or functional) N3 (motor* or movement* or neurological) N3 (disorder* or symptom*)) ) )

S3 TI ( (((disorder# or reaction or Hysteria or Neuros#s) N3 Conversion) or Astasia#Abasia) ) OR AB ( (((disorder# or reaction or Hysteria or Neuros#s) N3 Conversion) or Astasia#Abasia) )

S4 DE "Hysteria"

S5 TI ( ((Hysterical N1 Neuros#s) or Hysteria) ) OR AB ( ((Hysterical N1 Neuros#s) or Hysteria) )

S6 DE "Movement Disorders"

S7 TI ( ("dyskinesia syndrome#" or "etat marbre" or "status marmoratus") ) OR AB ( ("dyskinesia syndrome#" or "etat marbre" or "status marmoratus") )

S8 DE "Nervous System Disorders" AND DE "Psychology"

S9 DE "Somatoform Disorders"

S10 TI ( ((psychophysiological or psychosomatic) N3 disorder#) ) OR AB ( ((psychophysiological or psychosomatic) N3 disorder#) )

S11 TI ( ((disorder# or hysteria# or reaction#) N3 dissociative) ) OR AB ( ((disorder# or hysteria# or reaction#) N3 dissociative) )

S12 S1 OR S2 OR S3 OR S4 OR S5 OR S6 OR S7 OR S8 OR S9 OR S10 OR S11

S13 TI Interprofessional Relations OR AB Interprofessional Relations OR DE "Interdisciplinary Treatment Approach" OR TI Physician-Nurse Relations OR AB Physician-Nurse Relations

S14 TI ( (((Interprofessional or Inter-professional or interpersonal or inter-personal or interinstitutional or inter-institutional) N3 relation*) or (Etiquette N1 Medical)) ) OR AB ( (((Interprofessional or Inter-professional or interpersonal or inter-personal or interinstitutional or inter-institutional) N3 relation*) or (Etiquette N1 Medical)) )

S15 TI ( (multidisciplin* or multi-disciplin* or interdisciplin* or inter-disciplin* or transdisciplin* or trans-disciplin* or multiprofession* or multi-profession* or interprofession* or inter-profession* or transprofession* or trans-profession* or multispecialty or multi-specialty or multimodal* or multi-modal* or multifacet* or multi-facet* or multioccupation* or multi-occupation* or interoccupation* or inter-occupation*) ) ) OR AB ( ( (multidisciplin* or multi-disciplin* or interdisciplin* or inter-disciplin* or transdisciplin* or trans-disciplin* or multiprofession* or multi-profession* or interprofession* or inter-profession* or transprofession* or trans-profession* or multispecialty or multi-specialty or multimodal* or multi-modal* or multifacet* or multi-facet* or multioccupation* or multi-occupation* or interoccupation* or inter-occupation*) ) )

S16 TI ( ((collaborat* or coordinat* or co-ordinat* or cooperative or co-operative or continu* or shared or network or joint or integrat* or stepped or systematic or augment* or enhance*) N3 (approach or care or team* or health* or work* or interven* or service* or model* or effort* or manag*)) ) OR AB ( ((collaborat* or coordinat* or co-ordinat* or cooperative or co-operative or continu* or shared or network or joint or integrat* or stepped or systematic or augment* or enhance*) N3 (approach or care or team* or health* or work* or interven* or service* or model* or effort* or manag*)) )

S17 DE "Patient Centered Care"

S18 TI ( (('Health Care' or Healthcare or health or care) N3 Team#) ) OR AB ( (('Health Care' or Healthcare or health or care) N3 Team#) )

S19 DE "Treatment Planning" OR Patient Care Planning OR DE "Health Care Delivery"

S20 TI ( ((patient or nursing or goal#) N3 care) ) OR AB ( ((patient or nursing or goal#) N3 care) )

S21 TI ( ((system# or 'health care') N3 integrated) ) OR AB ( ((system# or 'health care') N3 integrated) )

S22 DE "Cooperative Education" OR Comprehensive Health Care

S23 TI ( (((Cooperative or Compliant) N3 Behaviors) or (Comprehensive N3 Care)) ) OR AB ( (((Cooperative or Compliant) N3 Behaviors) or (Comprehensive N3 Care)) )

S24 (S13 OR S14 OR S15 OR S16 OR S17 OR S18 OR S19 OR S20 OR S21 OR S22 OR S23)

S25 S12 AND S24

S26 DE "Placebo" OR DE "Randomized Controlled Trials" OR DE "Randomized Clinical Trials" OR DE "Experiment Controls"

S27 TI ("random*" OR "sham" OR "placebo*" OR "Nonrandom*" OR "non random*" OR "non-random*" OR "quasi-random*" OR "quasirandom*" OR "pragmatic study" OR "pragmatic studies") OR AB ("random*" OR "sham" OR "placebo*" OR "Nonrandom*" OR "non random*" OR "non-random*" OR "quasi-random*" OR "quasirandom*" OR "pragmatic study" OR "pragmatic studies") OR SU ("random*" OR "sham" OR "placebo*" OR "Nonrandom*" OR "non random*" OR "non-random*" OR "quasi-random*" OR "quasirandom*" OR "pragmatic study" OR "pragmatic studies") OR KW ("random*" OR "sham" OR "placebo*" OR "Nonrandom*" OR "non random*" OR "non-random*" OR "quasi-random*" OR "quasirandom*" OR "pragmatic study" OR "pragmatic studies")

S28 TI ((("singl*" OR "doubl*" OR "tripl*" OR "trebl*") W0 ("blind*" OR "dumm*" OR "mask*"))) OR AB ((("singl*" OR "doubl*" OR "tripl*" OR "trebl*") W0 ("blind*" OR "dumm*" OR "mask*"))) OR SU ((("singl*" OR "doubl*" OR "tripl*" OR "trebl*") W0 ("blind*" OR "dumm*" OR "mask*"))) OR KW ((("singl*" OR "doubl*" OR "tripl*" OR "trebl*") W0 ("blind*" OR "dumm*" OR "mask*")))

S29 TI ("control*" N2 ("study" OR "studies" OR "trial*" OR "group*")) OR AB ("control*" N2 ("study" OR "studies" OR "trial*" OR "group*")) OR KW ("control*" N2 ("study" OR "studies" OR "trial*" OR "group*"))

S30 TI "allocated" OR AB "allocated" OR SU "allocated"

S31 TI ((("open label" OR "open-label") N4 ("study" OR "studies" OR "trial*"))) OR AB ((("open label" OR "open-label") N4 ("study" OR "studies" OR "trial*"))) OR SU ((("open label" OR "open-label") N4 ("study" OR "studies" OR "trial*"))) OR KW ((("open label" OR "open-label") N4 ("study" OR "studies" OR "trial*")))

S32 TI ((("equivalence" OR "superiority" OR "non-inferiority" OR "noninferiority" OR "quasiexperimental" OR "quasi-experimental") N2 ("study" OR "studies" OR "trial*"))) OR AB ((("equivalence" OR "superiority" OR "non-inferiority" OR "noninferiority" OR "quasiexperimental" OR "quasi-experimental") N2 ("study" OR "studies" OR "trial*"))) OR SU ((("equivalence" OR "superiority" OR "non-inferiority" OR "noninferiority" OR "quasiexperimental" OR "quasi-experimental") N2 ("study" OR "studies" OR "trial*"))) OR KW ((("equivalence" OR "superiority" OR "non-inferiority" OR "noninferiority" OR "quasiexperimental" OR "quasi-experimental") N2 ("study" OR "studies" OR "trial*")))

S33 TI (("pragmatic" OR "practical") N2 "trial*") OR AB (("pragmatic" OR "practical") N2 "trial*") OR SU (("pragmatic" OR "practical") N2 "trial*") OR KW (("pragmatic" OR "practical") N2 "trial*")

S34 TI (("phase" N2 ("III" OR "3")) N2 ("study" OR "studies" OR "trial*")) OR SU (("phase" N2 ("III" OR "3")) N2 ("study" OR "studies" OR "trial*")) OR KW (("phase" N2 ("III" OR "3")) N2 ("study" OR "studies" OR "trial*"))

S35 S26 OR S27 OR S28 OR S29 OR S30 OR S31 OR S32 OR S33 OR S34

S36 S25 AND S35

S37 (DE "Meta Analysis" OR DE "Systematic Review" OR DE "Technology, Medical/EV" OR PT "systematic review" OR PT "meta analysis" OR (((TI systematic* OR AB systematic*) N3 ((TI review* OR AB review*) OR (TI overview* OR AB overview*))) OR ((TI methodologic* OR AB methodologic*) N3 ((TI review* OR AB review*) OR (TI overview* OR AB overview*)))) OR (((TI quantitative OR AB quantitative) N3 ((TI review* OR AB review*) OR (TI overview* OR AB overview*) OR (TI synthes* OR AB synthes*))) OR ((TI research OR AB research) N3 ((TI integrati* OR AB integrati*) OR (TI overview* OR AB overview*)))) OR (((TI integrative OR AB integrative) N3 ((TI review* OR AB review*) OR (TI overview* OR AB overview*))) OR ((TI collaborative OR AB collaborative) N3 ((TI review* OR AB review*) OR (TI overview* OR AB overview*))) OR ((TI pool* OR AB pool*) N3 (TI analy* OR AB analy*))) OR ((TI "data synthes*" OR AB "data synthes*") OR (TI "data extraction*" OR AB "data extraction*") OR (TI "data abstraction*" OR AB "data abstraction*")) OR ((TI handsearch* OR AB handsearch*) OR (TI "hand search*" OR AB "hand search*")) OR ((TI "mantel haenszel" OR AB "mantel haenszel") OR (TI peto OR AB peto) OR (TI "der simonian" OR AB "der simonian") OR (TI dersimonian OR AB dersimonian) OR (TI "fixed effect*" OR AB "fixed effect*") OR (TI "latin square*" OR AB "latin square*")) OR ((TI "met analy*" OR AB "met analy*") OR (TI metanaly* OR AB metanaly*) OR (TI "technology assessment*" OR AB "technology assessment*") OR (TI HTA OR AB HTA) OR (TI HTAs OR AB HTAs) OR (TI "technology overview*" OR AB "technology overview*") OR (TI "technology appraisal*" OR AB "technology appraisal*")) OR ((TI "meta regression*" OR AB "meta regression*") OR (TI metaregression* OR AB metaregression*)) OR (TI meta-analy* OR TI metaanaly* OR TI "systematic review*" OR TI "biomedical technology assessment*" OR TI "bio-medical technology assessment*" OR AB meta-analy* OR AB metaanaly* OR AB "systematic review*" OR AB "biomedical technology assessment*" OR AB "bio-medical technology assessment*" OR MW meta-analy* OR MW metaanaly* OR MW "systematic review*" OR MW "biomedical technology assessment*" OR MW "bio-medical technology assessment*") OR ((TI medline OR AB medline OR MW medline) OR (TI cochrane OR AB cochrane OR MW cochrane) OR (TI pubmed OR AB pubmed OR MW pubmed) OR (TI medlars OR AB medlars OR MW medlars) OR (TI embase OR AB embase OR MW embase) OR (TI cinahl OR AB cinahl OR MW cinahl)) OR (SO Cochrane OR SO health technology assessment OR SO evidence report) OR ((TI comparative OR AB comparative) N3 ((TI efficacy OR AB efficacy) OR (TI effectiveness OR AB effectiveness))) OR ((TI "outcomes research" OR AB "outcomes research") OR (TI "relative effectiveness" OR AB "relative effectiveness")) OR (((TI indirect OR AB indirect) OR (TI "indirect treatment" OR AB "indirect treatment") OR (TI mixed-treatment OR AB mixed-treatment) OR (TI bayesian OR AB bayesian)) N3 (TI comparison* OR AB comparison*)) OR ((TI multi* OR AB multi*) N3 (TI treatment OR AB treatment) N3 (TI comparison* OR AB comparison*)) OR ((TI mixed OR AB mixed) N3 (TI treatment OR AB treatment) N3 ((TI meta-analy* OR AB meta-analy*) OR (TI metaanaly* OR AB metaanaly*))) OR (TI "umbrella review*" OR AB "umbrella review*") OR ((TI multi* OR AB multi*) N2 (TI paramet* OR AB paramet*) N2 (TI evidence OR AB evidence) N2 (TI synthesis OR AB synthesis)) OR ((TI multiparamet* OR AB multiparamet*) N2 (TI evidence OR AB evidence) N2 (TI synthesis OR AB synthesis)) OR ((TI multi-paramet* OR AB multi-paramet*) N2 (TI evidence OR AB evidence) N2 (TI synthesis OR AB synthesis))

S38 S25 AND S37

Date 16/10/2025: **studies retrieved = 298**

**Cinahl (10/2025)**

S1 (MH "Somatoform Disorders+")

S2 TI ( ((psychogenic or functional) N3 (motor* or movement* or neurological) N3 (disorder* or symptom*)) ) OR AB ( ((psychogenic or functional) N3 (motor* or movement* or neurological) N3 (disorder* or symptom*)) )

S3 TI ( (((disorder# or reaction or Hysteria or Neuros#s) N3 Conversion) or Astasia#Abasia) ) OR AB ( (((disorder# or reaction or Hysteria or Neuros#s) N3 Conversion) or Astasia#Abasia) )

S4 (MH "Hysteria")

S5 TI ( ((Hysterical N1 Neuros#s) or Hysteria) ) OR AB ( ((Hysterical N1 Neuros#s) or Hysteria) )

S6 (MH "Movement Disorders")

S7 TI ( ("dyskinesia syndrome#" or "etat marbre" or "status marmoratus") ) OR AB ( ("dyskinesia syndrome#" or "etat marbre" or "status marmoratus") )

S8 (MH "Nervous System Diseases/PF")

S9 (MH "Psychophysiologic Disorders")

S10 TI ( ((psychophysiological or psychosomatic) N3 disorder#) ) OR AB ( ((psychophysiological or psychosomatic) N3 disorder#) )

S11 TI ( ((disorder# or hysteria# or reaction#) N3 dissociative) ) OR AB ( ((disorder# or hysteria# or reaction#) N3 dissociative) )

S12 S1 OR S2 OR S3 OR S4 OR S5 OR S6 OR S7 OR S8 OR S9 OR S10 OR S11

S13 (MH "Interprofessional Relations+") OR "Interdisciplinary Communication" OR (MH "Nurse-Physician Relations")

S14 TI ( (((Interprofessional or Inter-professional or interpersonal or inter-personal or interinstitutional or inter-institutional) N3 relation*) or (Etiquette N1 Medical)) ) OR AB ( (((Interprofessional or Inter-professional or interpersonal or inter-personal or interinstitutional or inter-institutional) N3 relation*) or (Etiquette N1 Medical)) )

S15 TI ( (multidisciplin* or multi-disciplin* or interdisciplin* or inter-disciplin* or transdisciplin* or trans-disciplin* or multiprofession* or multi-profession* or interprofession* or inter-profession* or transprofession* or trans-profession* or multispecialty or multi-specialty or multimodal* or multi-modal* or multifacet* or multi-facet* or multioccupation* or multi-occupation* or interoccupation* or inter-occupation*) ) OR AB ( (multidisciplin* or multi-disciplin* or interdisciplin* or inter- ...

S16 TI ( ((collaborat* or coordinat* or co-ordinat* or cooperative or co-operative or continu* or shared or network or joint or integrat* or stepped or systematic or augment* or enhance*) N3 (approach or care or team* or health* or work* or interven* or service* or model* or effort* or manag*)) ) OR AB ( ((collaborat* or coordinat* or co-ordinat* or cooperative or co-operative or continu* or shared or network or joint or integrat* or stepped or systematic or augment* or enhance*) N3 (approach or car ...

S17 (MH "Multidisciplinary Care Team+")

S18 TI ( (('Health Care' or Healthcare or health or care) N3 Team#) ) OR AB ( (('Health Care' or Healthcare or health or care) N3 Team#) )

S19 (MH "Patient Care") OR "patient care management" OR (MH "Patient Care Plans") OR "Patient Care Planning" OR (MH "Health Care Delivery, Integrated")

S20 TI ( ((patient or nursing or goal#) N3 care) ) OR AB ( ((patient or nursing or goal#) N3 care) )

S21 TI ( ((system# or 'health care') N3 integrated) ) OR AB ( ((system# or 'health care') N3 integrated) )

S22 (MH "Cooperative Behavior") OR "Comprehensive Health Care"

S23 TI ( (((Cooperative or Compliant) N3 Behavior#) or (Comprehensive N3 Care)) ) OR AB ( (((Cooperative or Compliant) N3 Behavior#) or (Comprehensive N3 Care)) )

S24 (S13 OR S14 OR S15 OR S16 OR S17 OR S18 OR S19 OR S20 OR S21 OR S22 OR S23)

S25 S12 AND S24

S26 (TI (systematic* N3 review*)) or (AB (systematic* N3 review*)) or (TI (systematic* N3 bibliographic*)) or (AB (systematic* N3 bibliographic*)) or (TI (systematic* N3 literature)) or (AB (systematic* N3 literature)) or (TI (comprehensive* N3 literature)) or (AB (comprehensive* N3 literature)) or (TI (comprehensive* N3 bibliographic*)) or (AB (comprehensive* N3 bibliographic*)) or (TI (integrative N3 review)) or (AB (integrative N3 review)) or (JN “Cochrane Database of Systematic Reviews”) or (TI ...

S27 S25 AND S26

S28 (MH "Clinical Trials+") OR PT Clinical trial OR TX clinic* n1 trial* OR TX (singl* n1 blind*) or (singl* n1 mask*) ) or TX ( (doubl* n1 blind*) or (doubl* n1 mask*) ) or TX ( (tripl* n1 blind*) or (tripl* n1 mask*) ) or TX ( (trebl* n1 blind*) or (trebl* n1 mask*) OR TX randomi* control* trial* OR (MH "Random Assignment" OR TX random* allocat* OR TX placebo* OR (MH "Placebos") OR (MH "Quantitative Studies") OR TX allocat* random*

S29 S25 AND S28

S30 S27 OR S29 Limiters - Exclude MEDLINE records

Date 16/10/2025: **studies retrieved = 192**
