# Supplementary material for: Interdisciplinary care models for functional movement disorders: a systematic review of clinical effectiveness and implications for integrated healthcare delivery
Source: Front Med (Lausanne). 2026 Oct 9;13:1939816. doi: 10.3389/fmed.2026.1939816 (PMC13690528; doi:10.3389/fmed.2026.1939816)
Supplement: Supplementary file 2 [file Table2.docx]

**Supplementary File 2.**

List of references that were excluded from the systematic search in the databases during the last screening stage, along with reasons for exclusion (n = 84).

| **Reason 1. Wrong study design (n = 36)** | |
| --- | --- |
| 1. | Bailey C, Tamasauskas A, Bradley-Westguard A, et al. What are the experiences of people with motor and sensory functional neurological disorder? A systematic review and thematic synthesis of qualitative studies. Disabil Rehabil 2025;47(1):1-15. |
| 2. | Amsden G. Managing psychogenic movement disorders with multidisciplinary approach: the best move. Ther. Perspect 2025;21, 14-15. |
| 3. | Aybek S, Hubschmid M, Mossinger C, Berney A, Vingerhoets F. Early intervention for conversion disorder: neurologists and psychiatrists working together. Acta Neuropsychiatr. 2013 Feb;25(1):52-6. |
| 4. | Aybek S, Lidstone SC, Nielsen G, MacGillivray L, Bassetti CL, Lang AE, Edwards MJ. What Is the Role of a Specialist Assessment Clinic for FND? Lessons From Three National Referral Centers. J Neuropsychiatry Clin Neurosci. 2020 Winter;32(1):79-84. |
| 5. | Bass C. Rehabilitation in Patients with Psychogenic Movement Disorders. Psychogenic movement disorders: Neurology and neuropsychiatry. 2006;():311-318 |
| 6. | Czarnecki K, Thompson JM, Seime R, Geda YE, Duffy JR, Ahlskog JE. Functional movement disorders: successful treatment with a physical therapy rehabilitation protocol. Parkinsonism Relat Disord 2012;18(3):247-251. |
| 7. | Dahlhauser, S. E., Theuer, A., & Hollman, J. (2017). Satisfaction and Occupational Performance in Patients with Functional Movement Disorder. The Open Journal of Occupational Therapy, 5(2). |
| 8. | Espay AJ, Aybek S, Carson A, et al. Current Concepts in Diagnosis and Treatment of Functional Neurological Disorders. JAMA Neurol 2018;75(9):1132-1141. |
| 9. | Faul L, Knight LK, Espay AJ, Depue BE, LaFaver K. Neural activity in functional movement disorders after inpatient rehabilitation. Psychiatry Res Neuroimaging. 2020 Sep 30;303:111125. |
| 10. | Gandolfi M, Sandri A, Mariotto S, Tamburin S, Paolicelli A, Fiorio M, Pedrotti G, Barone P, Pellecchia MT, Erro R, Cuoco S, Carotenuto I, Vinciguerra C, Botto A, Zenere L, Canu E, Sibilla E, Filippi M, Sarasso E, Agosta F, Tinazzi M; Group Collaborators Consortium. A window into the mind-brain-body interplay: Development of diagnostic, prognostic biomarkers, and rehabilitation strategies in functional motor disorders. PLoS One. 2024 Sep 26;19(9):e0309408. |
| 11. | Grubbs L, Rommelfanger KS. Placebo for psychogenic disorders: diagnosis, stigma, and treatment narratives. In: Johnson LSM, Rommelfanger KS, editors. The Routledge handbook of neuroethics. 1st ed. New York: Routledge; 2017. p. 103–120. |
| 12. | Hinson VK, Haren WB. Psychogenic movement disorders. Lancet Neurol. 2006 Aug;5(8):695-700. |
| 13. | Issak S, Kanaan R, Nielsen G, Fini NA, Williams G. Functional Gait Disorders: Clinical presentations, Phenotypes and Implications for treatment. Brain Inj. 2023 Apr 16;37(5):437-445. |
| 14. | Jacob AE, Kaelin DL, Roach AR, Ziegler CH, LaFaver K. Motor Retraining (MoRe) for Functional Movement Disorders: Outcomes From a 1-Week Multidisciplinary Rehabilitation Program. PM R. 2018 Nov;10(11):1164-1172. |
| 15. | Kola S, LaFaver K. Updates in Functional Movement Disorders: from Pathophysiology to Treatment Advances. Curr Neurol Neurosci Rep. 2022 May;22(5):305-311. |
| 16. | Lopez MR, Mordecai K, Blanken AE, LaFrance WC Jr. Caring for Patients With Functional Neurological Disorder-A New Paradigm. Cogn Behav Neurol. 2024 Mar 1;37(1):1-2. |
| 17. | Maggio J, Kyle K, Stephen CD, Perez DL. Lessons Learned in Outpatient Physical Therapy for Motor Functional Neurological Disorder. J Neurol Phys Ther. 2023 Jan 1;47(1):52-59. |
| 18. | Matin N, Young SS, Williams B, LaFrance WC Jr, King JN, Caplan D, Chemali Z, Weilburg JB, Dickerson BC, Perez DL. Neuropsychiatric Associations With Gender, Illness Duration, Work Disability, and Motor Subtype in a U.S. Functional Neurological Disorders Clinic Population. J Neuropsychiatry Clin Neurosci. 2017 Fall;29(4):375-382. |
| 19. | O'Neal MA, Baslet GC, Polich GR, Raynor GS, Dworetzky BA. Functional Neurologic Disorders: The Need for a Model of Care. Neurol Clin Pract. 2021 Apr;11(2):e152-e156. |
| 20. | Pepper E, Mohan A, Butcher K, Parsons M, Curtis J. Functional neurological disorders: an Australian interdisciplinary perspective. Med J Aust. 2022 Jun 6;216(10):501-503. |
| 21. | Perez DL, Edwards MJ, Nielsen G, Kozlowska K, Hallett M, LaFrance WC Jr. Decade of progress in motor functional neurological disorder: continuing the momentum. J Neurol Neurosurg Psychiatry. 2021 Mar 15:jnnp-2020-323953. |
| 22. | Petrie D, Lehn A, Barratt J, Hughes A, Roberts K, Fitzhenry S, Gane E. How Is Functional Neurological Disorder Managed in Australian Hospitals? A Multi-Site Study Conducted on Acute Inpatient and Inpatient Rehabilitation Wards. Mov Disord Clin Pract. 2023 Mar 28;10(5):774-782. |
| 23. | Polich G, Zalanowski S, Maney J, Perez DL, Baslet G, Maggio J, O'Neal MA, Dworetzky B, Herman S. Development of an inpatient rehabilitation pathway for motor functional neurological disorders: Initial reflections. NeuroRehabilitation. 2022;50(2):231-243. |
| 24. | Whiting DL, Riashi M, Nicholson Perry K. A clinician's quick guide of evidence-based approaches: functional neurological symptom disorder (conversion disorder). *Clin Psychol*. 2019;23(2):177–179. |
| 25. | Polich G, Thompson J, Molton I, Herman S, LaFaver K. Intensive rehabilitation for functional motor disorders (FMD) in the United States: A review. NeuroRehabilitation. 2022;50(2):245-254. |
| 26. | Fobian A. Establishment of an interdisciplinary functional neurological disorder (FND) treatment program and development of a clinical care pathway for FND [Internet]. ClinicalTrials.gov; 2024 [cited 2026 May 5]. Available from: <https://clinicaltrials.gov/study/NCT06362161>. Identifier: NCT06362161. |
| 27. | Ratcliffe S. Clinical briefing: functional neurological symptom disorder. *Br J Neurosci Nurs*. 2017;13(5):211–219. |
| 28. | Ricciardi L, Edwards MJ. Treatment of functional (psychogenic) movement disorders. Neurotherapeutics. 2014 Jan;11(1):201-7. |
| 29. | Richardson M, Isbister G, Nicholson B. A Novel Treatment Protocol (Nocebo Hypothesis Cognitive Behavioural Therapy; NH-CBT) for Functional Neurological Symptom Disorder/Conversion Disorder: A Retrospective Consecutive Case Series. Behav Cogn Psychother. 2018 Jul;46(4):497-503. |
| 30. | Richardson M, Kleinstäuber M, Wong D. Nocebo-Hypothesis Cognitive Behavioral Therapy (NH-CBT) for Persons With Functional Neurological Symptoms (Motor Type): Design and Implementation of a Randomized Active-Controlled Trial. Front Neurol. 2020 Dec 7;11:586359. |
| 31. | Zasler ND. Review of Functional movement disorder: An interdisciplinary case-based approach (LaFaver, Maurer, Nicholson, & Perez, 2022). NeuroRehabilitation. 2023;52(2):153-154. |
| 32. | Schmidt T, Ebersbach G, Oelsner H, Sprock A, König IR, Bäumer T, Münchau A, Weissbach A. Evaluation of Individualized Multi-Disciplinary Inpatient Treatment for Functional Movement Disorders. Mov Disord Clin Pract. 2021 Jun 24;8(6):911-918. |
| 33. | Shapiro AP, Teasell RW. Behavioural interventions in the rehabilitation of acute v. chronic non-organic (conversion/factitious) motor disorders. Br J Psychiatry. 2004 Aug;185:140-6. |
| 34. | Stephen, C.D., Schneider, D., Williams, D.T. (2022). Functional Movement Disorders. In: Frucht, S.J. (eds) Movement Disorder Emergencies. Current Clinical Neurology. Humana, Cham. |
| 35. | Tolchin B, Baslet G, Carson A, Dworetzky BA, Goldstein LH, LaFrance WC Jr, Martino S, Perez DL, Reuber M, Stone J, Szaflarski JP. The role of evidence-based guidelines in the diagnosis and treatment of functional neurological disorder. Epilepsy Behav Rep. 2021 Oct 21;16:100494. |
| 36. | Tsui P, Deptula A, Yuan DY. Conversion Disorder, Functional Neurological Symptom Disorder, and Chronic Pain: Comorbidity, Assessment, and Treatment. Curr Pain Headache Rep. 2017 Jun;21(6):29. |
| **Reason 2. Wrong patient population (n = 18)** | |
| 1. | Butler M, Seynaeve M, Nicholson TR, et al. Psychedelic treatment of functional neurological disorder: a systematic review. Ther Adv Psychopharmacol 2020;10:2045125320912125. |
| 2. | Beal EM, Coates P, Pelser C. Psychological interventions for treating functional motor symptoms: A systematic scoping review of the literature. Clin Psychol Rev 2022;94:102146. |
| 3. | Connors MH, Quinto L, Deeley Q, Halligan PW, Oakley DA, Kanaan RA. Hypnosis and suggestion as interventions for functional neurological disorder: A systematic review. Gen Hosp Psychiatry 2024;86:92-102. |
| 4. | Gonsalvez I, Spagnolo P, Dworetzky B, Baslet G. Neurostimulation for the treatment of functional neurological disorder: a systematic review. Epilepsy Behav Rep. 2021;16:100501. |
| 5. | Jackson K, Hamilton S, Jones S, Barr S. Patient reported experiences of using community rehabilitation and/or support services whilst living with a long-term neurological condition: a qualitative systematic review and meta-aggregation. Disabil Rehabil. 2019 Nov;41(23):2731-2749. |
| 6. | South West London and St George's Mental Health NHS Trust. Eye movement desensitisation and reprocessing therapy (EMDR) for functional neurological disorder (FND) (MODIFI) [Internet]. ClinicalTrials.gov; 2025 [cited 2026 May 5]. Available from: <https://clinicaltrials.gov/study/NCT05455450>. Identifier: NCT05455450. |
| 7. | Centre hospitalier de l’Université de Montréal (CHUM). Clinical evaluation of HRV biofeedback in functional neurological disorders compared to placebo (HRV_BFB_FND) [Internet]. ClinicalTrials.gov; 2024 [cited 2026 May 5]. Available from: <https://clinicaltrials.gov/study/NCT06422819>. Identifier: NCT06422819. |
| 8. | Oriuwa C, Mollica A, Feinstein A, Giacobbe P, Lipsman N, Perez DL, Burke MJ. Neuromodulation for the treatment of functional neurological disorder and somatic symptom disorder: a systematic review. J Neurol Neurosurg Psychiatry. 2022 Mar;93(3):280-290. |
| 9. | Patwal R, Jolly AJ, Kumar A, Yadav R, Desai G, Thippeswamy H. Diagnostic accuracy of clinical signs and investigations for functional weakness, sensory and movement disorders: A systematic review. J Psychosom Res. 2023 May;168:111196. |
| 10. | Pick S, Anderson DG, Asadi-Pooya AA, Aybek S, Baslet G, Bloem BR, Bradley-Westguard A, Brown RJ, Carson AJ, Chalder T, Damianova M, David AS, Edwards MJ, Epstein SA, Espay AJ, et al. Outcome measurement in functional neurological disorder: a systematic review and recommendations. J Neurol Neurosurg Psychiatry. 2020 Jun;91(6):638-649. |
| 11. | Poole NA, Wuerz A, Agrawal N. Abreaction for conversion disorder: systematic review with meta-analysis. Br J Psychiatry. 2010 Aug;197(2):91-5. |
| 12. | Schönfeldt-Lecuona C, Lefaucheur JP, Lepping P, Liepert J, Connemann BJ, Sartorius A, Nowak DA, Gahr M. Non-Invasive Brain Stimulation in Conversion (Functional) Weakness and Paralysis: A Systematic Review and Future Perspectives. Front Neurosci. 2016 Mar 31;10:140. |
| 13. | Bailey C, Tamasauskas A, Bradley-Westguard A, et al. What are the experiences of people with motor and sensory functional neurological disorder? A systematic review and thematic synthesis of qualitative studies. Disabil Rehabil 2025;47(1):1-15. |
| 14. | Cabreira V, McLoughlin C, Shivji N, Lodge A, Rhijn SV, Keynejad RC, Coebergh J, Carson A, Stone J, Lehn A, Hoeritzauer I. Functional neurological disorder in pregnancy, labour and the postpartum period: systematic review. BJPsych Bull. 2025 Dec;49(6):401-411. |
| 15. | Connors MH, Quinto L, Deeley Q, Halligan PW, Oakley DA, Kanaan RA. Hypnosis and suggestion as interventions for functional neurological disorder: A systematic review. Gen Hosp Psychiatry 2024;86:92-102. |
| 16. | Butler M, Seynaeve M, Nicholson TR, et al. Psychedelic treatment of functional neurological disorder: a systematic review. Ther Adv Psychopharmacol 2020;10:2045125320912125. |
| 17. | Daum C, Hubschmid M, Aybek S. The value of 'positive' clinical signs for weakness, sensory and gait disorders in conversion disorder: a systematic and narrative review. J Neurol Neurosurg Psychiatry 2014;85(2):180-190. |
| 18. | Sharpe M, Walker J, Williams C, Stone J, Cavanagh J, Murray G, Butcher I, Duncan R, Smith S, Carson A. Guided self-help for functional (psychogenic) symptoms: a randomized controlled efficacy trial. Neurology. 2011 Aug 9;77(6):564-72. |
| **Reason 3. Wrong outcomes (n = 2)** | |
| 1. | Dunalska A, Saramak K, Śmiłowska K, et al. Biomarkers of functional movement disorders - a systematic review. Neurol Neurochir Pol 2025;59(4):354-374. |
| 2. | Ortega-Robles E, Shalash A, Ramírez-Bermúdez J, Arias-Carrión O. Functional Movement Disorder: Evolving Mechanisms, Diagnostic Precision, and Personalized Multidisciplinary Care. Mov Disord Clin Pract 2025;12(9):1244-1258. |
| **Reason 4. Wrong intervention (n = 16)** | |
| 1. | Nielsen G, Marston L, Hunter RM, et al. Outcomes of specialist physiotherapy for functional motor disorder: the Physio4FMD RCT. Health Technol Assess 2025;29(34):1-28. |
| 2. | Sandri A, Di Vico IA, Geroin C, et al. Does duration Matter? Evaluating the impact of short- and long-term telemedicine in functional motor disorders. Parkinsonism Relat Disord 2025;137:107948. |
| 3. | O'Mahony B, Nielsen G, Baxendale S, Edwards MJ, Yogarajah M. Economic Cost of Functional Neurologic Disorders: A Systematic Review. Neurology. 2023 Jul 11;101(2):e202-e214. |
| 4. | Exposure therapy for functional neurological disorders [Internet]. Australian New Zealand Clinical Trials Registry (ANZCTR); 2022 [cited 2026 May 5]. Available from: https://www.anzctr.org.au/Trial/Registration/TrialReview.aspx?ACTRN=12622001464752. Identifier: ACTRN12622001464752. |
| 5. | Implementing a Digital Telerehabilitation Protocol and Non-motor Outcomes, and Quality of Life in Patients With Functional Motor Disorders: a Feasibility 2-arm Parallel Randomized Controlled Clinical Trial. 2024. |
| 6. | Gelauff JM, Rosmalen JGM, Carson A, Dijk JM, Ekkel M, Nielsen G, Stone J, Tijssen MAJ. Internet-based self-help randomized trial for motor functional neurologic disorder (SHIFT). Neurology. 2020 Sep 29;95(13):e1883-e1896. |
| 7. | Gutkin M, McLean L, Brown R, Kanaan RA. Systematic review of psychotherapy for adults with functional neurological disorder. J Neurol Neurosurg Psychiatry. 2020 Nov 5:jnnp-2019-321926. |
| 8. | Torbate-Heidaria University of Medical Sciences. Comparative of dialectic therapy and mindfulness-based cognitive therapy (MBCT) on reduction of anxiety disorder and conversion disorder in patients with heart-like symptoms [Internet]. Iranian Registry of Clinical Trials; 2022 [cited 2026 May 5]. Available from: <http://en.irct.ir/trial/64933>. Identifier: IRCT20181022041421N2. |
| 9. | McWhirter L, Ludwig L, Carson A, McIntosh RD, Stone J. Transcranial magnetic stimulation as a treatment for functional (psychogenic) upper limb weakness. J Psychosom Res. 2016 Oct;89:102-6. |
| 10. | Millman LSM, Williams IA, Jungilligens J, Pick S. Neurocognitive performance in functional neurological disorder: A systematic review and meta-analysis. Eur J Neurol. 2025 Jan;32(1):e16386. |
| 11. | Molero-Mateo P, Molina-Rueda F. Physiotherapy for patients with functional movement disorder: a systematic review. Neurologia (Engl Ed). 2024 Jul-Aug;39(6):505-514. |
| 12. | Tinazzi M, Università di Verona. Telemedicine in functional motor disorder (TeleFMD) [Internet]. ClinicalTrials.gov; 2022 [cited 2026 May 5]. Available from: <https://clinicaltrials.gov/study/NCT05345340>. Identifier: NCT05345340. |
| 13. | Nielsen G, Buszewicz M, Stevenson F, Hunter R, Holt K, Dudziec M, Ricciardi L, Marsden J, Joyce E, Edwards MJ. Randomised feasibility study of physiotherapy for patients with functional motor symptoms. J Neurol Neurosurg Psychiatry. 2017 Jun;88(6):484-490. |
| 14. | Nielsen G, Stone J, Edwards MJ. Physiotherapy for functional (psychogenic) motor symptoms: a systematic review. J Psychosom Res. 2013 Aug;75(2):93-102. |
| 15. | Tinazzi M. Effectiveness of telemedicine on health outcomes (clinical, humanistic, economic) in the management of patients with functional motor disorder: randomised controlled clinical trial [Internet]. ClinicalTrials.gov; 2022 [cited 2026 May 5]. Available from: <https://clinicaltrials.gov/study/NCT05345340>. Identifier: NCT05345340. |
| 16. | Torbate-Heidaria University of Medical Sciences. Comparison of DBT and MBCT on reduction of anxiety disorder and conversion disorder comparing dialectical therapy and mindfulness on reduction of anxiety disorder and conversion disorder [Internet]. Iranian Registry of Clinical Trials; 2022 [cited 2026 May 5]. Available from: <http://en.irct.ir/trial/64933>. Identifier: IRCT20181022041421N2. |
| **Reason 5. Language (n = 6)** | |
| 1. | Cras P, Crosiers D. [Psychogenic movement disorders]. Tijdschr Psychiatr 2015;57(2):104-108. |
| 2. | Huang Q, Wang G. Diagnosis and treatment of functional myoclonus. Chin J Contemp Neurol Neurosurg. 2023;23(11):1026–1030. |
| 3. | Popkirov S, Jungilligens J, Michaelis R. Funktionelle Bewegungsstörungen verstehen und verständlich machen. Nervenarzt. 2024 Jun;95(6):499. |
| 4. | Lu SY, Yang YW, Liu Y, Wang G, Xie Q, Wang JX. Advances on rehabilitation treatment of functional motor disorder. *Chin J Contemp Neurol Neurosurg*. 2023;23(12):1097–1102. |
| 5. | Li SH, Su W, Chen HB. Diagnosis and treatment of functional dystonia. Chin J Contemp Neurol Neurosurg. 2023;23(9):844–849. |
| 6. | Popkirov S, Jungilligens J, Michaelis R. Understanding and explaining functional movement disorders. Nervenarzt. 2024 Jun;95(6):499-506. German. |
| **Reason 6. Clinical trial ongoing (n = 3)** | |
| 1. | Efficacy of the multidisciplinary treatment on the quality of life of patients with functional movement disorders [Internet]. ClinicalTrials.gov; 2023 [cited 2026 May 5]. Available from: <https://clinicaltrials.gov/study/NCT05634486>. Identifier: NCT05634486. |
| 2. | University Hospital, Toulouse. rTMS effect in functional psychogenic movement disorders applied alone then combined with hypnotherapy (MOVHYSTIM) [Internet]. ClinicalTrials.gov; 2020 [cited 2026 May 5]. Available from: <https://clinicaltrials.gov/study/NCT02471066>. Identifier: NCT02471066. |
| 3. | Università di Verona. Digital telerehabilitation in functional motor disorders [Internet]. ClinicalTrials.gov; 2024 [cited 2026 May 5]. Available from: <https://clinicaltrials.gov/study/NCT06274281>. Identifier: NCT06274281. |
| **Reason 7. Communications (n = 2)** | |
| 1. | O´Mahony B. Yogarajah M. The economic cost of functional neurological disorders: A systematic review (P14-11.009). Neurology Letters to the Editor 2025 |
| 2. | Serranova T. Managing functional movement disorders. *Eur J Neurol*. 2019;26:967. doi:10.1111/ene.14021. |
| **Reason 8. Letter to the editor (n = 1)** | |
| 1. | Dallocchio C, Tinazzi M, Bombieri F, Arnó N, Erro R. Cognitive Behavioural Therapy and Adjunctive Physical Activity for Functional Movement Disorders (Conversion Disorder): A Pilot, Single-Blinded, Randomized Study. Psychother Psychosom 2016;85(6):381-383. |
