# Supplementary material for: Interdisciplinary care models for functional movement disorders: a systematic review of clinical effectiveness and implications for integrated healthcare delivery
Source: Front Med (Lausanne). 2026 Oct 9;13:1939816. doi: 10.3389/fmed.2026.1939816 (PMC13690528; doi:10.3389/fmed.2026.1939816)
Supplement: Supplementary file 3 [file Table3.docx]

**Supplementary File 3.** Search Strategies for qualitative evidence and reasons for exclusion

**Ovid MEDLINE (11/2025)**

1. exp "Conversion Disorder"/

2. ((psychogenic or functional) adj3 (motor* or movement* or neurological) adj3 (disorder* or symptom*)).ti,ab,kf.

3. (((disorder? or reaction or Hysteria or Neuros#s) adj3 Conversion) or Astasia?Abasia).tw.

4. Hysteria/

5. ((Hysterical adj1 Neuros?s) or Hysteria).tw.

6. Movement Disorders/

7. ("dyskinesia syndrome?" or "etat marbre" or "status marmoratus").tw.

8. Nervous System Diseases/px [Psychology]

9. Psychophysiologic Disorders/

10. ((psychophysiological or psychosomatic) adj3 disorder?).tw.

11. ((disorder? or hysteria? or reaction?) adj3 dissociative).tw.

12. 1 or 2 or 3 or 4 or 5 or 6 or 7 or 8 or 9 or 10 or 11

13. exp Interprofessional Relations/ or Interdisciplinary Communication/ or Physician-Nurse Relations/

14. (((Interprofessional or Inter-professional or interpersonal or inter-personal or interinstitutional or inter-institutional) adj3 relation*) or (Etiquette adj1 Medical)).tw,ab,kw.

15. (multidisciplin* or multi-disciplin* or interdisciplin* or inter-disciplin* or transdisciplin* or trans-disciplin* or multiprofession* or multi-profession* or interprofession* or inter-profession* or transprofession* or trans-profession* or multispecialty or multi-specialty or multimodal* or multi-modal* or multifacet* or multi-facet* or multioccupation* or multi-occupation* or interoccupation* or inter-occupation*).ti,ab,kw.

16. ((collaborat* or coordinat* or co-ordinat* or cooperative or co-operative or continu* or shared or network or joint or integrat* or stepped or systematic or augment* or enhance*) adj3 (approach or care or team* or health* or work* or interven* or service* or model* or effort* or manag*)).ti,ab,kw.

17. exp Patient Care Team/

18. (('Health Care' or Healthcare or health or care) adj3 Team?).tw. (39225)

19. patient care management/ or exp Patient Care Planning/ or exp "delivery of health care, integrated"/

20. ((patient or nursing or goal?) adj3 care).tw.

21. ((system? or 'health care') adj3 integrated).tw.

22. Cooperative Behavior/ or Comprehensive Health Care/

23. (((Cooperative or Compliant) adj3 Behaviors) or (Comprehensive adj3 Care)).tw.

24. 13 or 14 or 15 or 16 or 17 or 18 or 19 or 20 or 21 or 22 or 23

25. 12 and 24

26. (letter or "case report*" or "historical article*" or (comment or editorial or in vitro or news)).pt.

27. 25 not 26

28. ((("semi-structured" or semistructured or unstructured or informal or "in-depth" or indepth or "face-to-face" or structured or guide) adj3 (interview* or discussion* or questionnaire*)) or (focus group* or qualitative or ethnograph* or fieldwork or "field work" or "key informant")).ti,ab. or interviews as topic/ or focus groups/ or narration/ or qualitative research/

29. 27 and 28

30. animals/ not (animals/ and humans/)

31. 29 not 30

Date 17/11/2025: **studies retrieved = 106**

**Embase (11/2025)**

#1. 'conversion disorder'/exp

#2. ((psychogenic OR functional) NEAR/3 (motor* OR movement* OR neurological) NEAR/3 (disorder* OR symptom*)):ti,ab,kw

#3. (((disorder$ OR reaction OR hysteria OR neuros?s) NEAR/3 conversion):ti,ab) OR astasia$abasia:ti,ab

#4. 'hysteria'/de

#5. ((hysterical NEAR/1 neuros?s):ti,ab) OR hysteria:ti,ab

#6. 'motor dysfunction'/de

#7. "dyskinesia syndrome$":ti,ab OR 'etat marbre':ti,ab OR 'status marmoratus':ti,ab

#8. 'neurologic disease'/de AND 'psychology'/de

#9. 'psychosomatic disorder'/de

#10. ((psychophysiological OR psychosomatic) NEAR/3 disorder$):ti,ab

#11. ((disorder$ OR hysteria$ OR reaction$) NEAR/3 dissociative):ti,ab

#12. #1 OR #2 OR #3 OR #4 OR #5 OR #6 OR #7 OR #8 OR #9 OR #10 OR #11

#13. 'public relations'/exp OR 'interdisciplinary communication'/de OR 'doctor nurse relation'/de

#14. (((interprofessional OR 'inter professional' OR interpersonal OR 'inter personal' OR interinstitutional OR 'inter institutional') NEAR/3 relation*):ti,ab,kw) OR ((etiquette NEAR/1 medical):ti,ab,kw)

#15. multidisciplin*:ti,ab,kw OR 'multi disciplin*':ti,ab,kw OR interdisciplin*:ti,ab,kw OR 'inter disciplin*':ti,ab,kw OR transdisciplin*:ti,ab,kw OR 'trans disciplin*':ti,ab,kw OR multiprofession*:ti,ab,kw OR 'multi profession*':ti,ab,kw OR interprofession*:ti,ab,kw OR 'inter profession*':ti,ab,kw OR transprofession*:ti,ab,kw OR 'trans profession*':ti,ab,kw OR multispecialty:ti,ab,kw OR 'multi specialty':ti,ab,kw OR multimodal*:ti,ab,kw OR 'multi modal*':ti,ab,kw OR multifacet*:ti,ab,kw OR 'multi facet*':ti,ab,kw OR multioccupation*:ti,ab,kw OR 'multi occupation*':ti,ab,kw OR interoccupation*:ti,ab,kw OR 'inter occupation*':ti,ab,kw

#16. ((collaborat* OR coordinat* OR 'co ordinat*' OR cooperative OR 'co operative' OR continu* OR shared OR network OR joint OR integrat* OR stepped OR systematic OR augment* OR enhance*) NEAR/3 (approach OR care OR team* OR health* OR work* OR interven* OR service* OR model* OR effort* OR manag*)):ti,ab,kw

#17. 'patient care team'/exp

#18. (('health care' OR healthcare OR health OR care) NEAR/3 team$):ti,ab

#19. 'patient care'/de OR 'patient care planning'/exp OR 'integrated health care system'/exp

#20. ((patient OR nursing OR goal$) NEAR/3 care):ti,ab

#21. ((system$ OR 'health care') NEAR/3 integrated):ti,ab

#22. 'cooperation'/de OR 'health care'/de

#23. (((cooperative OR compliant) NEAR/3 behaviors):ti,ab) OR ((comprehensive NEAR/3 care):ti,ab)

#24. #13 OR #14 OR #15 OR #16 OR #17 OR #18 OR #19 OR #20 OR #21 OR #22 OR #23

#25. #12 AND #24

#26. 'conference abstract'/it OR 'conference paper'/it OR 'short survey'/it OR 'abstract report'/exp

#27. #25 NOT #26

#28. ((('semi-structured' OR semistructured OR unstructured OR informal OR 'in-depth' OR indepth OR 'face-to-face' OR structured OR guide) NEAR/3 (interview* OR discussion* OR questionnaire*)):ti,ab) OR (focus:ti,ab AND group*:ti,ab) OR qualitative:ti,ab OR ethnograph*:ti,ab OR fieldwork:ti,ab OR 'field work':ti,ab OR 'key informant':ti,ab OR 'interview'/exp OR 'information processing'/de OR 'verbal communication'/de OR 'qualitative research'/exp

#29. #27 AND #28

#30. #29 AND [embase]/lim NOT ([embase]/lim AND [medline]/lim) AND [humans]/lim

Date 17/11/2025: **studies retrieved = 116**

List of references that were excluded from the systematic search in the databases during the last screening stage, along with reasons for exclusion (n = 6).

| **Reason 1. Wrong patient population (n = 4)** | |
| --- | --- |
| 1. | McGovern E, Pringsheim T, Medina A, et al. Transitional Care for Young People with Neurological Disorders: A Scoping Review with A Focus on Patients with Movement Disorders. Mov Disord 2021;36(6):1316-1324. |
| 2. | Rezaei O, Stanley M. Understanding the lived experiences of individuals with functional neurological disorders (FND) in Australia. Disabil Rehabil 2025;47(24):6408-6415. |
| 3. | Sireci F, Moretti V, Cavallieri F, et al. "Somewhere Between an Actual Disease and a Disease": A Grounded Theory Study on Diagnosing Functional Neurological Disorders From a Multi-Informant Perspective. Qual Health Res 2024;34(11):1069-1083. |
| 4. | Szasz A, Korner A, McLean L. Qualitative systematic review on the lived experience of functional neurological disorder. BMJ Neurol Open 2025;7(1):e000694. |
| **Reason 2. Wrong intervention (n = 2)** | |
| 1. | Bailey C, Tamasauskas A, Bradley-Westguard A, et al. What are the experiences of people with motor and sensory functional neurological disorder? A systematic review and thematic synthesis of qualitative studies. Disabil Rehabil 2025;47(1):1-15. |
| 2. | Dal Pasquale BG, Teive HAG, von der Heyde MD, Dal Pasquale LFA. Management of Functional Seizures and Functional Movement Disorder: A Cross-Sectional Comparative Study. Neuropsychiatr Dis Treat 2022;18:2121-2131. |
