# Supplementary material for: Interdisciplinary care models for functional movement disorders: a systematic review of clinical effectiveness and implications for integrated healthcare delivery
Source: Front Med (Lausanne). 2026 Oct 9;13:1939816. doi: 10.3389/fmed.2026.1939816 (PMC13690528; doi:10.3389/fmed.2026.1939816)
Supplement: Supplementary file 4 [file Table4.docx]

**Supplementary File 4.**  Clinical efficacy of multidisciplinary interventions for individuals with functional movement disorders

| Author(s), year, | Study measures | | | | |
| --- | --- | --- | --- | --- | --- |
|  | Quality of life | Motor function | Mental health | Overall clinical assessment | Social impact |
| Macias-García et al, 2024, | EQ VAS:  *3 months (pre-post change):* MDG: 6.53 (CI 95%: -3.7 to 16.7) vs. CG: -5.53 (CI 95%: -12.8 to 1.7), p=0.07  *5 months:* MDG: 18.16 (CI 95%: 5.9 to 30.4) vs. CG: 3.05 (CI 95%: -1.7 to 29.0), p=0.6.  EQ Index^*^:  *3 months (pre-post change):* MDG: -0.01 (CI 95%: -0.1 to 0.1) vs. CG: -0.05 (CI 95%: -0.2 to 0.1), p=0.98  *5 months:* MDG: 0.06 (CI 95%: -0.1 to 0.2) vs. CG: -0.01 (CI 95%: -0.1 to 0.1), p=0.13  SF-36 PCS:  *3 months (pre-post change):* MDG: 2.35 (CI 95%: -0.6 to 5.3) vs. CG: -1.11 (CI 95%: -4.6 to 2.4), p=0.11  *5 months:* MDG: 3.21 (CI 95%: 0.3 to 6.1) vs. CG: -0.55 (CI 95%: -3.4 to 2.3), **p<0.001** | sFMDRS:  *3 months (pre-post change):* MDG: -7.11 (CI 95%: -9.6 to -4.6) vs CG: 0.26 (CI 95%: -1.3 to 1.9), **p<0.001**  *5 months:* MDG: -7.42 (CI 95%: -10.4 to -4.4) vs CG: 2.16 (CI 95%: 0.6 to 3.8), **p<0.001** | SF-36 MCS*:*  *3 months (pre-post change):* MDG: 1.19 (CI 95%: -3.9 to 6.4) vs. CG: 0.76 (CI 95%: -3.0 to 4.6), p=0.82  *5 months:* MDG: 3.07 (CI 95%: -2.4 to 8.6) vs. CG: 2.94 (CI 95%: -2.2 to 8.1), p=0.83 | CGI-S:  *3 months (pre-post change):* MDG: -1.32 (CI 95%: -1.8 to -0.8) vs. CG: 0.21 (CI 95%: -0.1 to 0.5), **p<0.001**  *5 months:* MDG: -1.53 (CI 95%: -2.0 a -1.1) vs. CG: 0.32 (CI 95%: -0.1 to 0.7), **p<0.001** | Zarit Caregiver Burden Interview:  *3 months (pre-post change):* MDG: -2.88 (CI 95%: -9.8 to 4.0) vs. CG: 4 (CI 95%: -2.7 to 10.7), p=0.94.  *5 months:* MDG: -0.29 (CI 95%: -7.1 to 6.5) vs. CG: 4.79 (CI 95%: -2.4 to 11.9), p=0.64 |
| Vizcarra et al, 2019, | ADL  *Pre-post change:* MDG: 0.7 (CI 95%: -0.4 to 1.8) vs. CG: 0.3 (CI 95%: -0.5 to 1.1), p=0.40  iADL  *Pre-post change:* MDG: 0.8 (CI 95%: -1.6 to 3.3) vs. CG: 0.3 (CI 95%: -0.5 to 1.1), p=0.57 | PMDRS  *Pre-post change:* MDG: -9.3 (CI 95%: -19.9 to 1.3) vs CG: -9 (CI 95%: -16.5 to -1.5), p=0.94 | HAM-D  *Pre-post change:* MDG: -6.8 (CI 95%: -18.2 to 4.5) vs. CG: -9.5 (CI 95%: -23.5 to 4.5), p=0.69  HAM-A  *Pre-post change:* MDG: -6.3 (CI 95%: -12.6 to -0.1) vs. CG: -5.5 (CI 95%: -24.8 to 13.8), p=0.91 | N/S | N/S |
| Hubchmid et al, 2015, | SF-36^‡^  *Basal:* 52% CG vs 46.5% MDG; p=0.60  *2 months:* 50.56% CG vs 43.57% MDG; p=0.53  *6 months:* 56.67% CG vs 37.14 % MDG; p=0.14  *12 months:* 65 % CG vs 53.57% MDG; p=0.38  Interaction time between group and treatment using MLM^§^ models: -0.265 (EE: 0.610), p=0.67 | *Rankin* ≥2^†^  *Basal:* CG: 81.82% vs. MDG: 70%; p=0.90  *2 months:* CG: 40% vs MDG: 44.44%; p=0.1  *6 months:* CG: 33.33% vs. MDG: 22.22%; p=1  *12 months:* CG: 12.5 % vs. MDG: 28.57%; p=0.90 | *SDQ-20* ≥ 30^†^  *Basal:* CG: 36.36% vs. MDG: 50%; p=0.85  *2 months:* CG: 40% vs. MDG:11.11%; p=0.36  *6 months:* CG: 22.22% vs. MDG: 12.5 %; p=1  *12 months follow-up:* CG: 37.5 % vs. MDG: 0%; p=0.24  Interaction time between group and treatment using MLM models ^§^: -0.524 (EE:0.217), **p<0.02**  *BDI* ≥ 20^†^  *Basal:* CG: 10% vs. MDG: 40%; p=0.30  *2 months:* CG: 22.22% vs. MDG: 28.57%; p=0.1  *6 months:* CG: 22.22% vs. MDG: 42.86%; p=0.73  *12 months:* CG: 28.57 % vs. MDG: 28.57%; p=1  Interaction time between group and treatment using the models of MLM^§^: -0.289 (EE: 0.127), **p<.05**  *MADRS* ≥ 15†  *Basal:* CG: 45.45% vs. MDG: 30%; p=0.78  *2 months:* CG: 40% vs. MDG: 44.44%; p=0.1  *6 months:* CG: 22.22% vs. MDG: 37.5%; p=0.88  *12 months:* CG: 28.57 % vs. MDG: 28.57%; p=1  Interaction time between group and treatment using MLM^§^ models: -0.014 *(*EE*:* 0.130), p=0.91  SF-36 MCS^‡^  *Basal:* CG: 64% vs. MDG: 54.93%; p=0.32  *2 months:* CG: 62.22% vs. MDG: 61.71%; p=0.97  *6 months:* CG: 63.11% vs. MDG: 65.14%; p=0.87  *12 months:* CG: 61.71 % vs. MDG: 69.71%; p=0.5  Interaction time between group and treatment using MLM§ models: 1.851 (EE: 0.775). **p<0.05** | *CGI* ≥4^†^  *Basal:* CG: 63.64% vs. MDG: 60%; p=0.1  *2 months:* CG: 30% vs. MDG: 66.67%; p=0.26  *6 months:* CG: 44.44% vs. MDG: 5.0 %; p=1  *12 months:* CG: 37.5 % vs. MDG: 28.57%; p=1  Interaction time between group and treatment using MLM^§^ models: -0.321 (EE: 0.134), p=0.02 | N/R |
| Jordbru et al, 2014, Norway | SF-12 Physical  *Basal:* MDG: 25.7 ± 8 vs. CG: 28.3 ± 8.6  *Post-treatment* ^#^*:* MDG: 37.2 ± 10.8 vs. CG: 27.3 ± 8.1, **p<0.001**  *12 months* ^¶^*:* 28.6 ±10.2 vs. CG: 44.5 ± 13.7  SF-12 Mental  *Basal:* MDG: 47.3 ±14.3 vs. CG: 42.9 ± 12.9  *Post-treatment* ^#^*:* MDG: 54.9 ± 9.0 vs. CG: 45.8 ±13.5, **p<0.001**  *12 months*^¶^*:* MDG: 49.3 ± 13.6 vs. CG: 52.1± 9.1 | FMS  *Basal:* MDG: 9.6 ± 4.3 vs. CG: 9.2 ±4.5  *Post-treatment* ^#^*:* MDG: 16.5 ± 4.6 vs. CG: 9.8 ±4.9; **p<0.001**  *12 months* ^¶^*:* MDG: 16.2 ± 4.5 vs. CG: 17.9 ± 0.4  FIM motor  *Basal:* MDG: 80.7 ± 12.2 vs. CG: 79.3 ± 13  *Post-treatment* ^#^*:* MDG: 90.1 ± 2.2 vs. CG: 80.9 ± 12.1; **p<0.001**  *12 months* ^¶^*:* MDG: 90.7 ± 0.7 vs. CG: 90.7 ± 0.7 | FIM cognitive  *Basal:* MDG: 34.45 ± 1.84 vs. CG: 35 ± 0  *Post-treatment* ^#^*:* MDG: 34.9 ± 0.2 vs. CG: 35 ± 0.0  *12 months* ^¶^*:* MDG: 35 ± 0.0 vs. CG: 35 ± 0.0 | N/R | N/R |
| Moene et al, 2002, Netherlands | ICIDH  *Basal:* MDG: 26.83 ± 9.8 vs. CG: 29.85 ± 10.77  *Post-treatment:* MDG: 23.17 ± 10.03 vs. CG: 23.55 ± 15.56  *8 months:* MDG: 20.78 ± 10.44 vs. CG: 18.80 ± 13.59  *Time-based condition:* p=0.26  ICIDHP  *Basal:* MDG: 26.71 ± 8.91 vs. CG: 28.20 ± 13.10  *Post-treatment:* MDG: 21.88 ± 11.44 vs. CG: 23.20 ± 16.61  *8 months:* MDG: 22.04 ± 11.94 vs. CG: 17.30 ± 14.29  *Time-based condition:* p=0.18 | VRMC  *Post-treatment ^**^*: MDG: 5.41 ± 1.44 vs. CG: 5.90 ± 1.28  *8 months:* MDG: 6.33 ± 1.13 vs. CG: 6.20 ± 1.20  *Time-based condition:* p=0.23 | SCL-90  *Basal:* MDG: 187.46 ± 54.20 vs. CG: 211.71 ± 67.95  *Post-treatment:* MDG: 181.75 ± 61.84 vs. CG: 180.33 ± 65.70  *8 months:* MDG: 176.74 ± 56.44 vs. CG: 182.71 ± 72.19  *Time-based condition:* p=0.16  SHCS  No significant correlations between pre-treatment SHCS scores and any of the outcome measures at post-treatment or at follow-up assessments. | N/R | N/R |

Abbreviations: CG: Control Group; MDG: Multidisciplinary treatment Group; MLM: Mixed Linear model; EE: Standard error; SD: Standard deviation; EQ-5D-5L (EQ Index): EuroQol 5 Dimensions 5 Levels; EQ VAS: EuroQol Visual Analogue Scale; SF-36: 36-Item Short-Form Survey; SF-12. Short Form-12 Health Survey: SF-36 MCS: Short-Form Survey Mental Component Summary; ADL. Activities of Daily Living Scale; iADL. Instrumental Activities of Daily Living Scale; ICIDH. International Classification of Impairments. Disabilities. and Handicaps Scale; ICIDHP. International Classification of Impairments. Disabilities. and Handicaps Scale Problems; sFMDRS. Simplified Functional Movement Disorders Rating Scale; PMDRS. Psychogenic Movement Disorders Rating Scale; FMS. Functional Mobility Scale; FIM. Functional Independence Measure; VRMC. The Video Rating Scale for Motor Conversion Symptoms; SDQ-20. Somatoform Dissociation Questionnaire; CGI. Clinical Global Impression Scale; CGI-S. Clinical Global Impression Scale- Severity; HAM-D. Hamilton Depression Rating Scale; HAM-A. Hamilton Anxiety Rating Scale; BDI. Beck Depression Inventory; MADRS. Montgomery and Asberg Depression Rating Scale; SCL-90. Symptom Checklist-90; SHCS. Stanford Hypnotic Clinical Scale; N/R, not reported

^*^ The EQ Index is a weighted score derived from the EQ-5D-5L, ranging from 0 (worst possible health status) to 1 (best possible health status, with no limitations in any dimension).

^†^ The results are expressed as the percentage of patients who meet or exceed the threshold specified for each tool.

^‡^ The results are shown as the mean and standard error.

^§^ The results are presented using MLM analysis, which aims to determine whether changes in outcomes over time differ between the treatment and control groups, in order to assess the efficacy of the study interventions.

^#^ The results are presented as means and standard deviations and refer to the end of treatment in the MDG group (3 weeks) and the end of the follow-up period in the CG group (4 weeks).

^¶^ The results are presented as means, and the standard deviation (SD) refers to the 12-month follow-up period for both the MDG and CG groups who received treatment, as this is a crossover study.

** No baseline values are reported.

Note: (n/s): results were not statistically significant. Bolt numbers are statistically significant results.

Note 2: p value in bold in the text are statistically significant.
