# Supplementary material for: Interdisciplinary care models for functional movement disorders: a systematic review of clinical effectiveness and implications for integrated healthcare delivery
Source: Front Med (Lausanne). 2026 Oct 9;13:1939816. doi: 10.3389/fmed.2026.1939816 (PMC13690528; doi:10.3389/fmed.2026.1939816)
Supplement: Supplementary file 5 [file Table5.docx]

**Supplementary File 5.** Quality of life assessment instruments used in the included studies

| Author(s), year | Quality of life assessment instruments | Patient assessment timeline |
| --- | --- | --- |
| Macías-García et al. 2024 | EQ-5D5L, SF-36 | T0, T1 (week 12),  T2 (week 20) |
| Vizcarra et al. 2019 | ADL, iADL | T0, T1 (week 12) |
| Hubschmid et al. 2015 | SF-36, work ability, demand or financial aid | T0, T1 (month 2), T2 (month 6), T3 (month 12) |
| Jordbru et al. 2014 | SF-12 | T0, T1 (month 1), T2 (month 12) |
| Moene et al. 2002 | ICIDH, ICIDHP | T0, T1 (month 1), T2 (month 12) |

**Abbreviations**: ADL= Katz index of independence in activities of daily living; EQ-5D-5L= EuroQol de 5-Dimensions 5-Levels; iADL=Lawton instrumental ADL; ICIDH= Impairments, Disabilities and Handicaps; ICIDHP= Impairments, Disabilities and Handicaps perceived problems; SF-12= 12-Item Short-Form Health Survey; SF-36=Medical Outcome Study 36-Item Short Form Health Survey.
